# The Insulin Receptor Adaptor IRS2 is an APC/C Substrate That Promotes Cell Cycle Protein Expression and a Robust Spindle Assembly Checkpoint

**DOI:** 10.1074/mcp.RA120.002069

**Published:** 2020-11-25

**Authors:** Sandhya Manohar, Qing Yu, Steven P. Gygi, Randall W. King

**Affiliations:** Department of Cell Biology, Harvard Medical School, Boston, Massachusetts, USA

**Keywords:** Anaphase-promoting complex/cyclosome, ubiquitin, cell cycle, insulin signaling pathway, G1, cell division, enzyme inhibition, mitosis, anaphase promoting complex (APC/C)

## Abstract

Insulin receptor substrate 2 (IRS2) is an essential adaptor that mediates signaling downstream of the insulin receptor and other receptor tyrosine kinases. Transduction through IRS2-dependent pathways is important for coordinating metabolic homeostasis, and dysregulation of IRS2 causes systemic insulin signaling defects. Despite the importance of maintaining proper IRS2 abundance, little is known about what factors mediate its protein stability. We conducted an unbiased proteomic screen to uncover novel substrates of the Anaphase Promoting Complex/Cyclosome (APC/C), a ubiquitin ligase that controls the abundance of key cell cycle regulators. We found that IRS2 levels are regulated by APC/C activity and that IRS2 is a direct APC/C target in G_1_. Consistent with the APC/C's role in degrading cell cycle regulators, quantitative proteomic analysis of IRS2-null cells revealed a deficiency in proteins involved in cell cycle progression. We further show that cells lacking IRS2 display a weakened spindle assembly checkpoint in cells treated with microtubule inhibitors. Together, these findings reveal a new pathway for IRS2 turnover and indicate that IRS2 is a component of the cell cycle control system in addition to acting as an essential metabolic regulator.

## Teaser

We report a chemical proteomic screen to uncover substrates of the APC/C, a ubiquitin ligase that controls cell division. We found that IRS2, a component of the insulin receptor signaling network that is required for normal metabolic homeostasis, is a direct APC/C substrate during G_1_. Analysis of IRS2 knockout cells revealed depletion of cell cycle regulatory proteins and a weakened spindle assembly checkpoint. Thus, IRS2 is a component of the cell cycle control system in addition to acting as a metabolic regulator.

## Graphical Abstract

**Figure:**
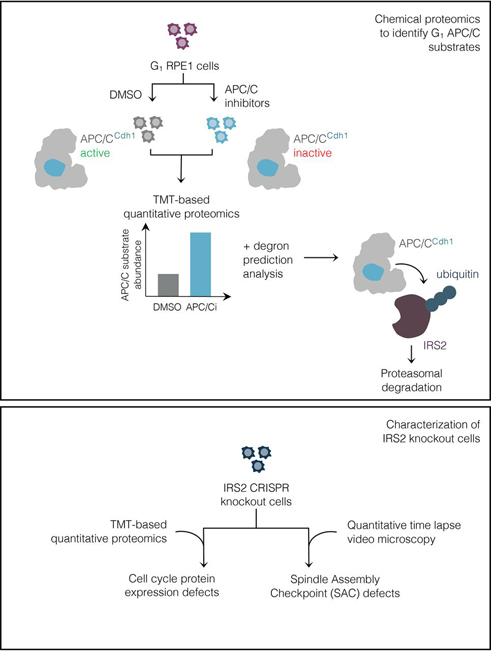

**Highlights**

• Chemical proteomics in G_1_ cells treated with small molecule APC/C inhibitors reveals novel putative APC/C substrates.
• The insulin receptor adaptor IRS2 is an APC/C substrate that is targeted for degradation by APC/C^Cdh1^.
• Quantitative proteomics in IRS2 knockout cells reveals a deficiency in cell cycle related protein expression.
• IRS2 promotes a robust spindle assembly checkpoint (SAC) during M-phase.

The insulin and insulin-like growth factor 1 receptors (IR/IGF1R) are receptor tyrosine kinases that control metabolism, differentiation, and growth. Upon ligand binding at the cell surface, the activated IR/IGF1R undergoes a conformational change that allows it to auto-phosphorylate tyrosine residues on its cytoplasmic subunits (1). This facilitates the recruitment and phosphorylation of insulin receptor substrate (IRS) proteins, which serve as scaffolds to initiate downstream signaling (2). Two major pathways that are stimulated by this cascade are the PI3K-AKT and Ras-Raf-MAPK pathways, which coordinate metabolic homeostasis and growth, among other functions (1).

The most physiologically important and ubiquitously expressed IRS proteins are IRS1 and IRS2. Though IRS1 and IRS2 share similar structural and functional features, they have complementary roles and expression patterns that depend on tissue type and physiological state (1). These differences are illustrated by divergent phenotypes in knockout mice: whereas IRS1 knockout mice exhibit insulin resistance that is compensated by increased pancreatic β cell mass, IRS2 knockout mice exhibit β cell failure and resultant diabetes (3). Distinct roles for IRS1 and IRS2 can also be observed within the same tissue. For example, in skeletal muscle, IRS1 is required for glucose uptake and metabolism, whereas IRS2 is important for lipid uptake and metabolism (4, 5). Furthermore, recent work has shown that the ratio of IRS1 to IRS2 is important for hepatic glucose metabolism (6). Thus, maintaining proper IRS1 and IRS2 levels is critical for systemic and cellular homeostasis.

The ubiquitin-mediated proteolysis of IRS proteins is important for restraining signaling through the IR/IGF1R. For example, both IRS proteins are targeted for proteasomal destruction following persistent insulin or IGF1 stimulation in a negative feedback loop that attenuates PI3K-AKT signaling (2, 7). In mice, removal of a ubiquitin ligase that is responsible for IGF1-induced degradation of IRS1 enhances insulin sensitivity and increases plasma glucose clearance (7). Though several ubiquitin ligases have been reported to control IRS1's proteasome-dependent degradation (8, 9, 10, 11, 12), only the Elongin BC complex in cooperation with SOCS1/3 have been implicated in driving IRS2 turnover (11). This is an intriguing disparity because hepatic IRS1 remains stable between fasting and feeding whereas IRS2 levels drop after feeding (13), suggesting that IRS2 is less stable than IRS1 in some physiological contexts. Because SOCS1/3 also targets IRS1, there are no reports of ubiquitin ligases that target IRS2 but not IRS1, leaving a gap in our knowledge of how IRS1 and IRS2 are differentially regulated by the ubiquitin proteasome system.

The Anaphase-Promoting Complex/Cyclosome (APC/C) is a 1.2 MDa ubiquitin ligase that targets key cell cycle-related proteins for destruction by the proteasome (14, 15). To transfer ubiquitin to its substrates, the APC/C works with one of two co-activators: Cdc20 during M-phase or Cdh1 during G_1_. These co-activators stimulate the catalytic activity of the APC/C and facilitate substrate recognition. APC/C^Cdc20^ and APC/C^Cdh1^ recognize substrates via short degron motifs in unstructured protein regions called destruction boxes (D-boxes) and KEN-boxes. An additional degron, called the ABBA motif, is only used by APC/C^Cdc20^ in metazoan cells (14, 15, 16).

Our laboratory has previously reported two small molecule inhibitors of APC/C activity: proTAME and apcin (17, 18). These inhibitors disrupt unique protein-protein interactions that are important for APC/C activity: proTAME binds the APC3 subunit, blocking co-activator loading, whereas apcin blocks the D-box recognition site on either Cdc20 or Cdh1. When used together in asynchronous cells, proTAME and apcin synergize to arrest cells in mitosis due to the blockade of APC/C^Cdc20^ activity and the subsequent stabilization of cyclin B1 (17). In contrast, APC/C inhibition in G_1_ cells blocks APC/C^Cdh1^ activity, which does not cause cell cycle arrest but causes the accumulation of Cdh1 substrates. To probe the substrate landscape of the APC/C, we conducted an unbiased proteomic screen by acutely blocking APC/C^Cdh1^ activity with small molecule APC/C inhibitors in G_1_ cells. Using this approach, we uncovered diverse putative APC/C^Cdh1^ substrates, including IRS2. We demonstrate that IRS2, but not IRS1, is a direct target of APC/C^Cdh1^, thereby establishing a novel mode by which IRS1 and IRS2 are differentially regulated. Using IRS2 knockout cell lines, we show that IRS2 is important for the expression of proteins involved in cell cycle progression. We further show that genetic deletion of IRS2 perturbs spindle assembly checkpoint function. Taken together, these data establish a role for IRS2 in normal cell cycle progression, revealing new connections between an essential component of the growth factor signaling network and cell cycle regulation.

## EXPERIMENTAL PROCEDURES

##### Cell Culture and Synchronization

All cell lines used in this work (HeLa, C_2_C_12_, hTERT-RPE1-FUCCI, HEK293T) were cultured in a humidified incubator at 37 °C in the presence of 5% CO_2_. HeLa, hTERT-RPE1 and C_2_C_12_ cells were obtained from American Type Culture Collection (ATCC), and hTERT-RPE1 cells were modified with FUCCI constructs (19) with the permission of the RIKEN Institute. HeLa cells were grown in DMEM with 10% FBS. Proliferating C_2_C_12_ myoblasts were grown in DMEM with 15% FBS, whereas differentiated myotubes were cultured in differentiation media, consisting of DMEM with 2% horse serum and 1× insulin, transferrin, selenium (ITS) Premix Universal Culture Supplement (Corning, 354350). hTERT-RPE1-FUCCI cells were grown in DMEM/F12 with 10% FBS supplemented with 0.01 mg/ml hygromycin B (Corning, 30-240-CR). HEK293T cells used for lentivirus generation were a gift from Wade Harper and were cultured in DMEM with 10% FBS. All cell lines tested were negative for mycoplasma contamination (Lonza LT07-218).

HeLa cells were synchronized by double thymidine block by treating with 2 mm thymidine for 18 h, releasing for 8 h, and re-treating with 2 mm thymidine for 19 h. HeLa cells synchronized by thymidine-nocodazole block were treated with 2 mm thymidine for 20 h, released for 8 h, then treated with 300–330 nm nocodazole for 15 h. Mitotic cells were collected by shake-off and re-plated in drug-free media for cell cycle time course experiments.

RPE1 cells were synchronized by RO3306 treatment by treating with 7.5 μm RO3306 for 18 h before releasing into fresh media for 30–40 min, after which cells were collected by mitotic shake-off and re-plated for cell cycle time course experiments. For G_1_ arrest experiments, RPE1 cells were treated with 1 μm palbociclib for 20 h.

To differentiate C_2_C_12_ myoblasts into myotubes, cells were grown to confluence and washed 2× in DMEM with 2% horse serum before switching to differentiation media. Cells were incubated for 72 h, with media changes every 24–36 h. Differentiation into myotubes was monitored visually as well as by immunoblotting for MyoD, a myogenic marker.

##### Immunoblotting

**Fig. 5:**
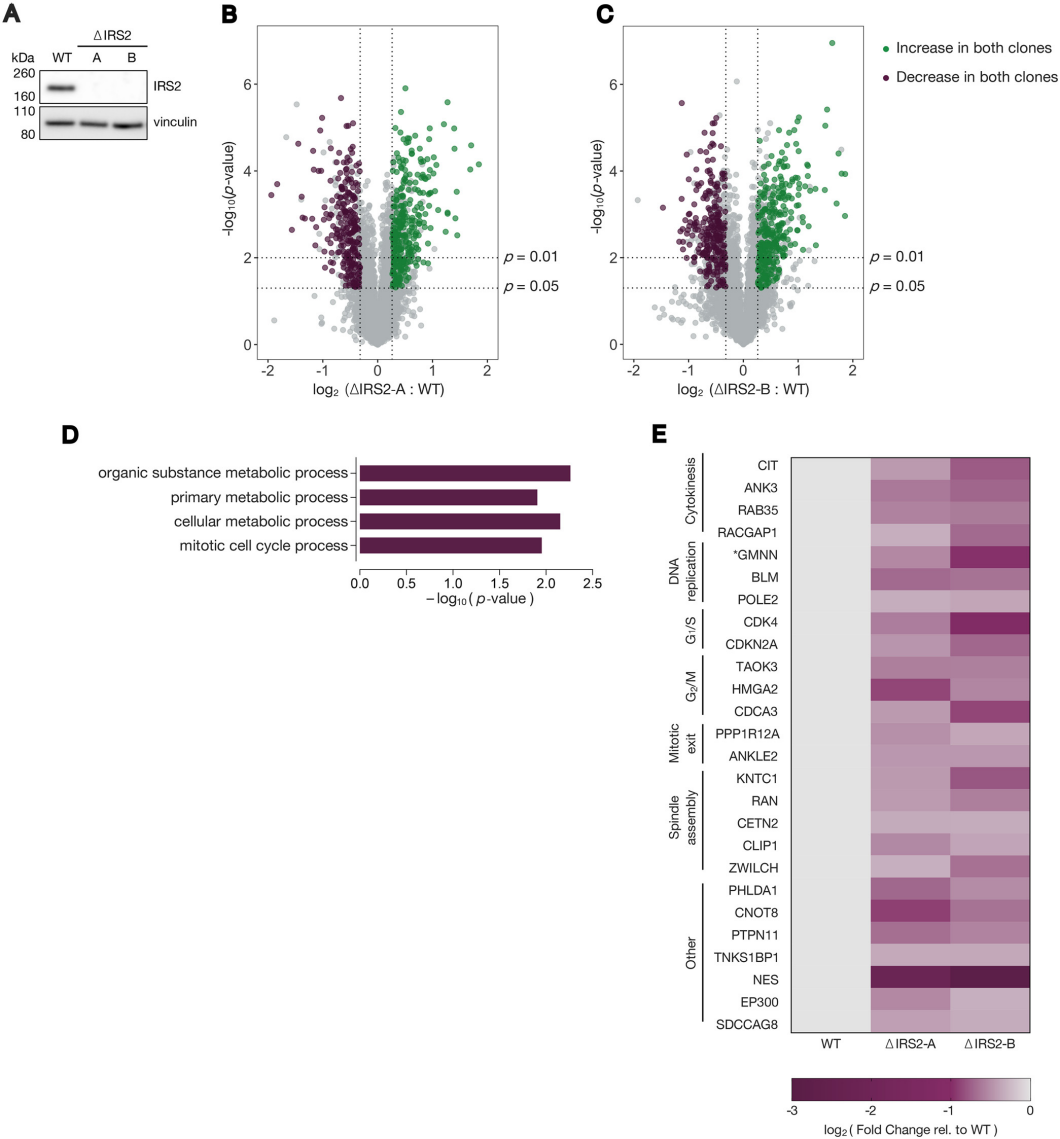
**IRS2 knockout cell lines are defective in mitotic cell cycle-related protein expression.***A*, WT, ΔIRS2-A, and ΔIRS2-B cell line lysates were analyzed for IRS2 expression by immunoblotting. *B–C*, Volcano plots comparing proteomes of ΔIRS2 cell lines with WT cell line. Proteins that significantly decrease > 20% (*p*-value < 0.05) in both cell lines compared with WT are shown in *purple*; proteins that significantly increase > 20% (*p*-value < 0.05) in both cell lines compared with WT are shown in *green*. *D*, Gene ontology (GO) term enrichment of proteins that decrease in both ΔIRS2 cell lines relative to WT cells. *E*, Heat map depicting cell cycle-related protein abundance changes between ΔIRS2 cell lines and WT cells. * : we could not distinguish whether the peptides from GMNN were from the endogenous protein or the mAG1-geminin (1-110) reporter.

Cell extracts were prepared in lysis buffer (10 mm Tris HCl pH 7.4, 100 mm NaCl, 1 mm EDTA, 1 mm EGTA, 1 mm NaF, 1 mm PMSF, 20 mm Na_4_P_2_O_7_, 2 mm NA_3_VO_4_, 1% Triton X-100, 10% glycerol, 0.1% SDS, and 0.5% deoxycholate) supplemented with Pierce protease inhibitor tablets (Thermo Fisher Scientific, A32963) and Pierce phosphatase inhibitor tablets (Thermo Fisher Scientific, A32957). Pellets were incubated in lysis buffer on ice for 30 min with vortexing and were centrifuged at 13,000rpm for 10 min to clear the lysate. Protein concentrations were determined using a bicinchoninic acid (BCA) assay (Thermo Fisher Scientific, 23225). Supernatants were re-suspended in NuPAGE LDS sample buffer (Thermo Fisher Scientific, NP0008) supplemented with 100 mm DTT (DTT) and boiled at 100 °C for 5 min. Equal masses of lysates were separated by SDS-PAGE using either 4–12% Bis Tris gels or 3–8% Tris acetate gels (Thermo Fisher Scientific). All IRS2 immunoblots were separated on 3–8% Tris acetate gels except for those shown in Fig. 5*A* and supplemental Fig. S4B, which were separated on 4–12% Bis Tris gels. Proteins were transferred to polyvinylidene difluoride (PVDF) membranes (Thermo Fisher Scientific, 88518).

Membranes were blocked in 5% nonfat dry milk in TBS with 0.1% Tween (TBS-T) before incubating with primary antibodies overnight at 4 °C with agitation. Membranes were probed with secondary antibodies dissolved in 5% milk in TBS-T for 1–2 h at room temperature before developing with an Amersham Pharmacia Biotech 600RGB imaging system. Quantification of immunoblots was done using ImageJ (20).

##### Antibodies

The following commercially available primary antibodies were used for immunoblotting: anti-IRS2 (Cell Signaling Technologies, 4502) 1:750; anti-Cdh1/Fzr1 (Sigma Aldrich, CC43) 1:500; anti-anillin (a gift from Christine Field (21)) 1:1000; anti-Aurora B (Bethyl, A300-431) 1:1000; anti-Eg5 (Cell Signaling Technologies, 4203) 1:1000; anti-Top2A (Cell Signaling Technologies, 12286) 1:1000; anti-TK1 (Cell Signaling Technologies, 8960) 1:1000; anti-Mps1 (Abcam, ab11108), 1:1000); anti-APC3 (BD Transduction Laboratories, 610455) 1:500; anti-cyclin B1 (Santa Cruz Biotechnology, sc-752) 1:500; anti-Cdc20 (Santa Cruz Biotechnology, sc-8358) 1:500; anti-c-Myc (9E10, Santa Cruz Biotechnology, sc-40) 1:1000; anti-HA-peroxidase (Sigma Aldrich), 1:1500; anti-cyclin A2 (Santa Cruz Biotechnology, sc-596) 1:500; anti-IRS1 (Cell Signaling Technologies, 2382) 1:750; anti-MyoD1 (Cell Signaling Technologies, 13812) 1:750; anti-GAPDH (Abcam, ab8245) 1:2000; anti-α tubulin (Abcam, ab7291 and Santa Cruz Biotechnology, sc-8035) 1:1000 for both; anti-vinculin (Santa Cruz Biotechnology, sc-73614) 1:2000. Secondary antibodies used: anti-rabbit IgG-HRP (GE Healthcare, NA934) and anti-mouse IgG-HRP (GE Healthcare, NA931V), both at 1:3000 dilutions.

##### Compounds

The following chemicals were used: palbociclib (LC Laboratories, P-7722), proTAME (Boston Biochem, I-440), MG132 (474790, Calbiochem), S-trityl l-cysteine (STLC, Alfa Aesar, L14384), thymidine (Sigma Aldrich, T9250), nocodazole (Sigma Aldrich, 31430-18-9), RO3306 (AdipoGen Life Sciences, AGCR13515M), doxycycline hyclate (Sigma Aldrich, D9891). Apcin was custom synthesized by Sundia MediTech Company (Lot #A0218-10069-031) using methods described previously (17). All compounds were dissolved in dimethyl sulfoxide (DMSO), with the exception of thymidine and doxycycline, which were dissolved in Dulbecco's PBS (DPBS, Corning, 21-030-CV). Dissolved compounds were stored at −20 °C before use.

##### CRISPR/Cas9 Mediated Gene Editing

A TrueGuide crRNA directed against exon 1 of Hs IRS2's coding region (target DNA sequence: 5‘- TCG AGA GCG ATC ACC CGT TT -3‘, Assay ID number: CRISPR850215_CR, Thermo Fisher Scientific) was annealed to the TrueGuide tracrRNA (Thermo Fisher Scientific, A35507) according to manufacturer protocol. hTERT RPE1-FUCCI cells were co-transfected with TrueCut Cas9 protein v2 (Thermo Fisher Scientific, A36496) and the annealed tracrRNA:crRNA complex using the LipofectAMINE CRISPRMAX Cas9 Transfection reagent (Thermo Fisher Scientific, CMAX00003) according to manufacturer protocol. Transfected cells were incubated for 2 days before switching to fresh media and expanding. Single cell clones were isolated using the limiting dilution method in a 96-well format, and clonal cell lines were expanded before screening for knockouts by immunoblotting.

##### Site Directed Mutagenesis

R777-E111 Hs.IRS2 and R777-E111 Hs.IRS2-nostop were gifts from Dominic Esposito (Addgene plasmid #70395 and #70396, respectively). Both of these plasmids encode codon optimized sequences for IRS2, with and without a stop codon respectively. R972A mutations were introduced into the aforementioned IRS2 clones using the Q5 Site-Directed Mutagenesis Kit (New England BioLabs) with the primers 5′ - AGA TTA TAT GAA TAA GTC CAC TGT CAG ATT ATA TG - 3′ and 5′ - GAC AGT GGA CTT GCC TGG CGA GAG TCT GAA CT - 3′ according to the manufacturer's protocol. For N-terminally FLAG-HA-tagged constructs, the insert from R77-E111 Hs.IRS2 (WT or) was cloned into the pHAGE-FLAG-HA-NTAP vector (a gift from Wade Harper) using the Gateway LR Clonase II system (Invitrogen). For doxycycline-inducible, C-terminally HA-tagged constructs, the insert from R77-E111 Hs.IRS2-nostop (WT or DM) was cloned into pINDUCER20 (a gift from Stephen Elledge, Addgene plasmid #44012) using the Gateway LR Clonase II system (Invitrogen). The DM mutation was verified both before and after Gateway cloning by Sanger sequencing.

##### Lentivirus Construction

To construct lentiviruses, HEK293T cells were co-transfected with pPAX2, pMD2, and either pINDUCER-20-IRS2 or pHAGE-FLAG-HA-NTAP-IRS2 in a 4:2:1 DNA ratio using LipofectAMINE 3000 (Invitrogen, L3000001) according to manufacturer's instructions. pPAX2 and pMD2 were gifts from Wade Harper. Twenty-four hours after transfection, HEK293T cells were switched to fresh media (DMEM + 10% FBS). Forty-eight hours after transfection, lentiviruses were harvested by clearing debris by centrifugation at 960 × *g* for 5 min and filtering through 0.45 μm SFCA filters. Lentiviruses were either used immediately or flash frozen in liquid nitrogen and stored at −80°C for later use.

##### Stable Cell Line Construction

To generate stable cell lines, plated HeLa, hTERT-RPE1, or C_2_C_12_ cells were incubated with lentiviruses and 2 µg/ml protamine sulfate. Twenty-four hours after viral infection, cells were switched to fresh media. Forty-eight hours after viral infection, antibiotics were introduced. For lentiviruses derived from pINDUCER20, geneticin (Invitrogen, 10131027) was used at a concentration of 750 µg/ml for both hTERT-RPE1 and C_2_C_12_ for 6–7 days. For lentiviruses derived from pHAGE-FLAG-HA-NTAP, puromycin (Sigma Aldrich, P8833) was used at a concentration of 0.5 µg/ml for 3 days. Antibiotic-selected populations of cells were expanded and used for further experiments without clonal selection.

##### Small Interfering RNAs (siRNAs)

Cells were transfected using RNAiMax (Invitrogen, 13778100) according to manufacturer's instructions with the following siRNAs: siGENOME NonTargeting Control siRNA #5 (d-001210-05, Dharmacon); ON-TARGETplus Human FZR1 siRNA (J-015377-08, Dharmacon), 25 nm; SMARTpool ON-TARGETplus Mouse Fzr1 siRNA (l-065289-01-0005), 25 nm. Cells were treated with siRNAs for 24 h for all experiments. For experiments involving subsequent compound treatment, cells were switched to fresh media before the addition of compounds.

##### Plasmid Transfection

C_2_C_12_ myoblasts were transfected with a plasmid containing the N-terminal 88 amino acids of human cyclin B1 fused to EGFP using Lipofectamine 3000 (Invitrogen, L3000001) with the P3000 reagent according to manufacturer's instructions. Growth media was refreshed to remove transfection reagents 24 h post-transfection, and cells were switched to differentiation media for an additional 3 days.

For ubiquitylation studies, HeLa cells were transfected with equal amounts of pCI-6xHis-hUb and/or pHAGE-NTAP-IRS2 using Lipofectamine 3000 with the P3000 reagent according to manufacturer's instructions. For myc-Cdh1 overexpression studies, HeLa cells were transfected with 0, 0.5, 1, and 2 μg of pCS2+ myc-hCdh1 using Lipofectamine 3000 with the P3000 reagent according to manufacturer's instructions.

##### In Vivo Ubiquitylation Assay

HeLa cells expressing pCI-6xHis-hUb and pHAGE-NTAP-IRS2 were treated with 10 μm MG132 either alone or in the presence of APC/C inhibitors (6 μm proTAME + 50 μm apcin) for 8 h. Following drug treatment, cells were collected, washed in Dulbecco's Phosphate Buffered Saline with calcium and magnesium (Corning, 21-030-CM), and flash frozen in liquid nitrogen. Cell pellets were lysed in denaturing lysis buffer (8M urea; 100 mm Na_2_HPO_4_; 0.05% Tween-20; 10 mm imidazole HCl, pH 8.0; 100 mm Tris HCl, pH 8.0; 1 × protease inhibitor mixture) by periodic vortexing on ice. Lysates were cleared at 13,000 rpm for 10 min Protein concentrations were measured by BCA assay. Equal amounts of protein were incubated with Ni-NTA agarose resin (Qiagen, 30210) for 4 h, rotating at 4 °C. Resins were washed three times in denaturing wash buffer (8M urea; 100 mm Na_2_HPO_4_; 0.05% Tween-20; 20 mm imidazole HCl, pH 8.0; 100 mm Tris HCl, pH 8.0) followed by three washes in native wash buffer (100 mm Na_2_HPO_4_; 0.05% Tween-20; 20 mm imidazole HCl, pH 8.0; 100 mm Tris HCl, pH 8.0). His-ubiquitin conjugates were eluted by boiling resin in 2× LDS sample buffer (Thermo Fisher Scientific, NP0008) supplemented with 200 mm DTT and 200 mm imidazole HCl, pH 8.0 for 10 min Input samples were resolved on 4–12% Bis-Tris gels (Thermo Fisher Scientific), and Ni-NTA elution samples were resolved on 3–8% Tris acetate gels (Thermo Fisher Scientific). Ponceau staining was used as a loading control.

##### Time Lapse and Fluorescence Microscopy

Cells were plated in a 24-well coverslip-bottom plate (Greiner BioOne, 662892). After 24 h, cells were treated with the indicated compounds and were imaged immediately afterward. Plates were inserted into a covered cage microscope incubator (Okolab) with temperature and humidity control at 37°C and 5% CO_2_ and mounted on a motorized microscope stage (Prior ProScan III). All images were collected on a Nikon Ti motorized inverted microscope equipped with a 20 ×/0.75 NA Plan Apo objective lens and the Perfect Focus system. mCherry fluorescence was excited with a Lumencor Spectra-X using a 555/25 excitation filter and a 605/52 emission filter (Chroma). mAG1 fluorescence was excited using a 490/20 excitation filter and a 525/36 emission filter (Chroma). Both configurations used a Sedat Quad dichroic (Chroma). Images were acquired with a Hamamatsu Orca-R2 or Hamamatsu Flash 4.0 V2 controlled with Nikon Elements image acquisition software. Three fields of view were collected per condition, and phase contrast and/or fluorescence images were captured at 5- to 8-min intervals (depending on the experiment) for 24–48 h.

Videos were analyzed using ImageJ. Mitotic duration was defined as the time from nuclear envelope breakdown (NEB) until division, death (cytoplasmic blebbing), or mitotic slippage. mAG1 and mCherry intensities were quantified manually by measuring the maximum intensity of signal for each cell in a given frame across multiple time points. For experiments analyzing fluorescence intensity during G_1_ arrest, measurements were made for all cells in a frame for each time point.

##### Experimental Design and Statistical Rationale

For G_1_ proteomics experiments, cells were treated with 1 μm palbociclib for 20 h at which point cells were either collection (t_0_) or treated with DMSO (control) or APC/C inhibitors (6 μm proTAME + 50 μm apcin) for an additional 8 h before collection (*n* = 3). For IRS2 knockout proteomic analysis, the proteomes of one control cell line (which had been subject to the CRISPR process but expressed WT levels of IRS2) and two distinct clonal IRS2 knockout lines were measured (*n* = 3). For both G_1_ APC/C inhibitor proteomics and IRS2 knockout cell line analysis, samples were analyzed in biological triplicate (*e.g.* from separate starter cultures) to increase statistical power. Technical replicates were not measured for either experiment. No samples were excluded from either analysis. Peptide spectral matches were filtered with a linear discriminant analysis (LDA) method to a 1% FDR (22) and a protein-level FDR of 1% was also implemented (23). To calculate protein-level FDR, the posterior probabilities reported for each peptide were multiplied to give a protein level probability estimate (22, 24). Using the Picked FDR method (23) proteins were filtered to the target 1% level. Subsequently, protein identifications were collapsed to a minimal number of identified proteins using the maximum parsimony principle. For both data sets, comparisons were calculated using a two-tailed, unpaired Student's *t*-test. For the G_1_ proteomics experiment, we considered significantly changing proteins as those with a *p*-value < 0.05 that increased ≥1.15-fold (based on the fold-changes observed for previously reported APC/C substrates within our data set). To justify our cutoff of a 1.15-fold change, we performed a power analysis for the t-tests that generated *p*-value < 0.05. The power analysis targeted a delta of 15% change, a significance level of 0.05, and a power of 0.95. The number of observations per group calculated to obtain the target has a median value of 2.28 (supplemental Fig. S2C). For the IRS2 knockout cell line analysis, we considered significantly changing proteins as those with a *p*-value < 0.05 and a <20% change in abundance. For experiments regarding the stability of IRS2-WT and IRS2-DM, *p*-values were calculated by two-way ANOVA. For fluorescence microscopy experiments that quantify mAG1 intensity in response to drug treatment over time, *p*-values were calculated by two-way ANOVA. For microscopy experiments that quantify mitotic duration following nocodazole treatment, *p*-values were calculated by one-way ANOVA. For time lapse microscopy data, *p*-values were calculated by one-way ANOVA. Gene enrichment was calculated using the AmiGO 2 search tool (25). Error bars represent standard deviation (S.D.) or standard error of the mean (S.E.) where indicated.

##### TMT Mass Spectrometry Sample Preparation

Cells were cultured as described in biological triplicate. Cells pellets were re-suspended in urea lysis buffer: 8M urea, 200 mm EPPS pH 8.0, Pierce protease inhibitor tablets (Thermo Fisher Scientific, A32963), and Pierce phosphatase inhibitor tablets (Thermo Fisher Scientific, A32957). Lysates were passed through a 21-gauge needle 20 times, and protein concentrations were measured by BCA assay (Thermo Fisher Scientific). One hundred micrograms of protein were reduced with 5 mm tris-2-carboxyethyl-phosphine (TCEP) at room temperature for 15 min, alkylated with 10 mm iodoacetamide at room temperature for 30 min in the dark, and were quenched with 15 mm DTT for 15 min at room temperature. Proteins were precipitated using a methanol/chloroform extraction. Pelleted proteins were resuspended in 100 µL 200 mm EPPS, pH 8.0. LysC (Wako, 125-05061) was added at a 1:50 enzyme/protein ratio, and samples were incubated overnight at room temperature with agitation. Following overnight incubation, trypsin (Promega, V5111) was added at a 1:100 enzyme/protein ratio, and samples were incubated for an additional 6 h at 37 °C. Tryptic digestion was halted by the addition of acetonitrile (ACN). Tandem mass tag (TMT) isobaric reagents (Thermo Fisher Scientific, 90406) were dissolved in anhydrous ACN to a final concentration of 20 mg/ml, of which a unique TMT label was added at a 2:1 label:peptide ratio. Peptides were incubated at room temperature for one hour with vortexing after 30 min TMT labeling reactions were quenched by the addition of 10 µL of 5% hydroxylamine. Equal amounts of each sample were combined at a 1:1 ratio across all channels and lyophilized by vacuum centrifugation. Samples were re-suspended in 1% formic acid (FA)/99% water and were desalted using a 50 mg 1 cc SepPak C18 cartridge (Waters, WAT054955) under vacuum. Peptides were eluted with 70% ACN/1% FA and lyophilized to dryness by vacuum centrifugation. The combined peptides were fractionated with basic pH reversed-phase (BPRP) HPLC, collected in a 96-well format and consolidated to a final of 24 fractions, out of which only alternating fractions (a total of 12) were analyzed (26). Each fraction was desalted via StageTip, lyophilized to dryness by vacuum centrifugation, and reconstituted in 5% ACN/5% FA for LC–MS/MS processing.

##### TMT Mass Spectrometry Analysis

Data for the G_1_ APC/C inhibition experiment were collected on an Orbitrap Fusion mass spectrometer coupled to a Proxeon EASY-nLC 1000 liquid chromatography (LC) pump (Thermo Fisher Scientific), whereas data for IRS2 knockout cell line analysis were collected on an Orbitrap Fusion Lumos mass spectrometer coupled to a Proxeon EASY-nLC 1200 liquid chromatography (LC) pump. The 100 μm capillary column was packed with 30 cm of Accucore 150 resin (2.6 μm, 150 Å; Thermo Fisher Scientific). Mobile phases were 5% ACN, 0.125% FA (Buffer A) and 95% ACN, 0.125% FA (Buffer B). Peptides from G_1_ APC/C inhibition experiment were separated using a 2.5 h gradient from 4% to 26% Buffer B and analyzed with a SPS-MS3 method (27). Peptides from IRS2 knockout cell line analysis were separated using a 2 h gradient from 4% to 30% Buffer B and analyzed with a real-time search strategy (28, 29). The MS proteomics data have been deposited to the ProteomeXchange Consortium via the PRIDE (30) partner repository with the data set identifier PXD018329 and 10.6019/PXD018329.

Raw data were converted to mzXML format using a modified version of RawFileReader (v3.0.77, https://planetorbitrap.com/rawfilereader) and searched with SEQUEST (v28 revision 12) (31) using an in-house proteomic pipeline against a human protein target-decoy database containing both SwissProt and TrEMBL entries (downloaded February 2014). The sequence database searched was the Human UniProt database (downloaded 02/04/2014), and 39,860 entries were searched including common contaminants (*e.g.* trypsin, human keratins). Searches were performed with a 50 ppm precursor mass tolerance, 0.9 Da fragment mass tolerance, trypsin digest with up to 2 missed cleavages. Allowed modifications include cysteine carboxyamidomethylation (+57.02146), static TMT on lysine and peptide N-temini (+229.16293) and up to 3 variable methionine oxidation (+15.99491). Peptide spectral matches were filtered with a linear discriminant analysis (LDA) method to a 1% FDR (22) and a protein-level FDR of 1% was also implemented (23). For peptide quantification, we extracted the TMT signal-to-noise and column normalized each channel to correct for equal protein loading. Peptide spectral matches with summed signal-to-noise less than 100 were excluded from final result. Lastly, each protein was scaled such that the summed signal-to-noise for that protein across all channels equals 100, thereby generating a relative abundance (RA) measurement.

##### High Content Mitotic Fraction Assay

Asynchronous RPE1-FUCCI WT or IRS2 knockout cell lines were plated in a black, clear-bottom 96-well plate (Corning, 3606). Plates were sealed with breathable white rayon sealing tape (Nunc, 241205) to prevent evaporation following plating and during all subsequent incubations. In experiments involving RNAi, cells were treated with siRNAs for 24 h. Cells were switched to fresh media, and compounds were added at the indicated concentrations for an additional 18 h. Following compound treatment, cells were fixed and stained directly without additional washing steps (to avoid the loss of loosely attached mitotic cells) with 10% formalin, 0.33 µg/mL Hoechst 33342, and 0.1% Triton X-100 in DPBS. Plates were sealed with aluminum tape (Nunc, 276014) and were incubated for 45 min room temperature in the dark before imaging. All experimental conditions were represented in triplicate on the same plate. Plates were imaged using an ImageXpress Micro high-content microscope (Molecular Devices) equipped with a 10× objective lens. Four images were acquired per well, yielding a total of 12 images per conditions. Images were processed automatically in ImageJ to identify and count nuclei as well as measure their maximum fluorescence intensity. ImageJ output files were pooled, and cumulative frequency curves for the maximum intensity of the cell population in each condition were computed using MATLAB. An intensity threshold was set based on the intensity of mitotic cells in control (DMSO-treated) wells to delineate interphase cells from mitotic cells. The fraction of mitotic cells was calculated as the fraction of cells above the set intensity threshold in MATLAB (17).

## RESULTS

##### Chemical Proteomics Reveals Proteins Whose Abundances Are APC/C^Cdh1^-Regulated

**Fig. 1:**
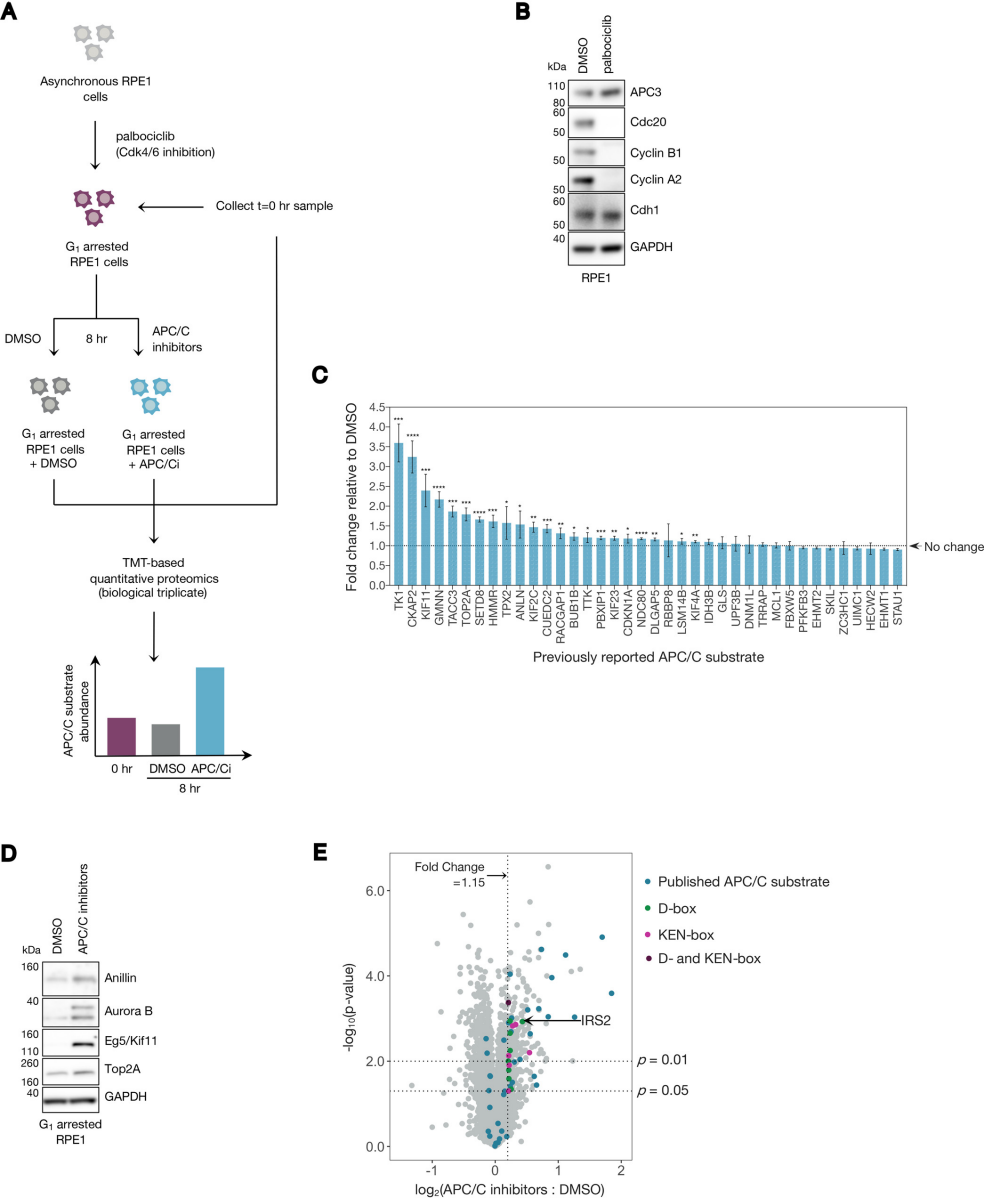
**High resolution chemical proteomics reveals proteins whose abundances are APC/C regulated.***A*, Workflow for the chemical proteomics experiment described in this study. Asynchronous RPE1 cells were arrested in 1 μm palbociclib (a Cdk4/6 inhibitor) for 20 h, at which point they were acutely treated with either DMSO or a combination of 6 μm proTAME + 50 μm apcin (referred to as “APC/C inhibitors” or “APC/Ci”), in the ongoing presence of palbociclib. Cells were then collected at time 0 (the time of APC/C inhibitor addition) or 8 h after drug addition and were harvested for TMT-based proteomic identification and quantification. Samples were analyzed in biological triplicate within a 10-plex TMT label set, with the 10th channel used as a bridge. *B*, Asynchronous RPE1 cells were treated with either DMSO or 1 μm palbociclib for 20 h. Cells were harvested, and lysates were analyzed by immunoblot for the indicated proteins. *C*, Previously reported APC/C substrates that were identified in this study are plotted with their observed fold change in the APC/C inhibitor treated sample (APC/Ci) relative to the DMSO treated sample. Error bars represent the standard deviation (S.D.) between the three biological replicates measured by MS. Asterisks indicate an abundance increase over control that is statistically significant (*: *p* < 0.05; **: *p* < 0.01; ***: *p* < 0.001; ****: *p* < 0.0001) *D*, Previously reported APC/C substrates that were identified as increasing by MS in G_1_ RPE1 cells treated with APC/C inhibitors were validated by immunoblot for selected proteins. *E*, Volcano plot highlighting all published APC/C substrates identified in this study (*blue*) as well as proteins that (1) contain a high probability D- and/or KEN-box (d-box = *green*, KEN-box = *pink*, D- and KEN-boxes = *purple*), (2) increase ≥1.15-fold under APC inhibition, (3) were identified by >1 peptide, and (4) have a *p*-value < 0.05.

To identify novel substrates and pathways regulated by APC/C^Cdh1^, we designed an experiment that coupled small molecule inhibition of the APC/C in G_1_ cells to high resolution tandem mass tag (TMT)-based quantitative proteomics (Fig. 1*A*). Blocking Cdk4/6 activity inhibits Rb phosphorylation, causing cells to arrest at the G_1_ restriction point (32). Thus, to generate a homogeneous population of G_1_ cells, we treated asynchronous hTERT-RPE1 cells bearing fluorescent ubiquitylation-based cell cycle indicator (FUCCI) constructs (19) with the Cdk4/6 inhibitor palbociclib. Our decision to conduct this analysis in RPE1 cells was guided by several factors. First, RPE1 cells are are Rb-positive and therefore palbociclib-sensitive. Second, their cell cycle dynamics are well-characterized, and they are easy to image. Finally, they are nontransformed with a near-diploid karyotype. Following G_1_ arrest, cells were acutely treated with a combination of APC/C inhibitors (6 μm proTAME + 50 μm apcin) or vehicle (DMSO) for 8 h. Cells were then collected for proteomic analysis with the expectation that APC/C^Cdh1^-regulated proteins would be stabilized in cells treated with APC/C inhibitors compared with control cells (Fig. 1*A*). The combined use of proTAME and apcin results in robust inhibition of the APC/C (17), which guided our decision to use this treatment scheme. Moreover, this scheme was designed to specifically identify APC/C^Cdh1^ substrates rather than APC/C^Cdc20^ substrates because APC/C^Cdh1^ degrades Cdc20 during G_1_ phase (33). Illustrating this point, Cdc20 expression was strongly reduced in G_1_ palbociclib-arrested cells (Fig. 1*B*). The expression of cyclins A and B was also reduced, consistent with a G_1_ block.

The experimental approach outlined in Fig. 1*A* was validated using the FUCCI reporter system. This system relies on the expression of two stably integrated fluorescent fusion proteins—mAG1-geminin (1–110) and mCherry-Cdt1 (30–120)—to monitor the activity of endogenous cell cycle-related ubiquitin ligases APC/C^Cdh1^ and SCF^Skp2^, respectively (19). As expected, cells treated with palbociclib showed a reduction in mAG1-geminin (1–110) fluorescence over time because of APC/C^Cdh1^ activity whereas mCherry-Cdt1 (30-120) intensity was increased, indicating G_1_ arrest (supplemental Figs. S1A–S1B> and supplemental Video S1). This arrest is reversible because removal of palbociclib causes cells to re-enter the cell cycle (supplemental Video S3). The addition of APC/C inhibitors in palbociclib-arrested cells rescued mAG1-geminin (1-110) levels (supplemental Figs. S1C–S1D and supplemental Video S2), confirming that this workflow stabilizes APC/C targets. Notably, cells released from palbociclib-mediated arrest accumulate mAG1-geminin (1–110) more rapidly than palbociclib-arrested cells treated with APC/C inhibitors (supplemental Fig. S1E), indicating that APC/C inhibition is likely insufficient to trigger cell cycle re-entry under these conditions. Consistent with this observation, no mitotic entry was observed over the duration of the experiment or when G1 arrested cells were treated with APC/C inhibitors for longer durations (up to 19 h) (supplemental Video S2).

Asynchronous RPE1 cells were arrested in G_1_ by treating them with palbociclib for 20 h. Cells were then either collected (time = 0 h) or treated with DMSO or APC/C inhibitors for an additional 8 h while maintaining a constant dose of palbociclib. We then measured relative protein abundances in biological triplicate using TMT-coupled quantitative proteomics (Fig. 1*A* and supplemental Table S1). We included the time = 0 h condition to monitor the effects of continued palbociclib arrest between the time of drug addition and the time of sample collection. Thus, all protein fold-changes discussed henceforth refer to changes between the 8 h APC/C inhibitor-treated and DMSO-treated samples. Notably, we detected 38 previously reported APC/C substrates in our data set (Figs. 1*C*–1*E*; supplemental Table S2). Of these, 22 increased significantly (*p* < 0.05) under conditions of APC/C inhibition relative to DMSO. We validated these findings in the context of several previously reported substrates by immunoblot (Fig. 1*D*). As an internal control, we detected a significant increase (*p* = 3.2 × 10^−5^) in the abundance of peptides derived from the N-terminal 110 amino acids of geminin (GMNN). These residues are shared with the mAG1-geminin (1–110) reporter expressed in this cell line, confirming earlier fluorescence-based validation of our experimental system.

We next developed a strategy to identify putative direct APC/C substrates from the set of proteins that were up-regulated in the presence of APC/C inhibitors. Of the 38 previously reported APC/C substrates that we identified, the median fold change under APC/C inhibition compared with DMSO was 1.15. Based on this, we screened for proteins that: (1) had a fold change ≥1.15 under APC/C inhibition (see Experimental Procedures and supplemental Fig. S2C for further analysis of the utility of this threshold in yielding statistically significant observations), (2) were identified and quantified based on >1 peptide, and (3) had a *p*-value < 0.05 across the three biological replicates measured in this experiment. This narrowed our analysis to a subset of 204 proteins (supplemental Table S3). Because many proteins that were up-regulated in the presence of APC/C inhibitors likely represent indirect effects of APC/C inhibition on gene expression, we employed another filtering strategy to identify direct APC/C targets based on the presence of known APC/C degron motifs. The APC/C recognizes substrates based on D-box motifs (RxxL or the extended motif RxxLxxxxN) and KEN-box motifs (KEN), so we used the SLiMSearch (Short Linear Motif Search) degron prediction tool (16, 34) to scan this 204-protein subset for proteins that contain these sequences. To classify a putative D- or KEN-box sequence as a probable physiological degron, we applied the following restrictions on the SLiMSearch (34) parameters: (1) similarity score ≥ 0.75; (2) consensus similarity is medium or high; (3) disorder score ≥ 0.4; (4) the putative degron must be intracellular and exist on a nonsecreted protein. These cutoffs were determined based on those met by previously validated APC/C substrates (including those not identified in our data set) and by the physical restriction that APC/C activity occurs within the cell. Based on these thresholds, our analysis identified 26 proteins as potential D- and KEN-box containing APC/C^Cdh1^ substrates (Table I, Fig. 1*E*). Of these 26 proteins, 11 have previously been reported as direct APC/C substrates, validating internally that this analysis was useful for identifying APC/C substrates.

**Table I:** 26 proteins containing high-probability D- and KEN-boxes as identified from G_1_ APC/C inhibitor proteomics. Features of the putative degron(s) found in each protein are annotated, including the SLiMSearch similarity score to other validated degrons, the similarity of the surrounding consensus sequence to other validated degrons, the disorder score for the region of the protein in which the degron is located, and the citation of the publication that reports the protein as an APC/C substrate, where applicable. *While DIAPH3/mDia2 has been shown to be ubiquitylated in a cell cycle dependent manner and was suggested as an APC/C substrate, there is no direct cell-based or biochemical evidence for this. **We cannot delineate whether the geminin peptides identified here derive from the FUCCI reporter or the endogenous protein. Proteins that have not been previously reported as APC/C substrates are shown in bold

Gene symbol	Fold change (APC/Ci : DMSO)	Similarity score	Disorder score	Reference (If Applicable)
D-box containing proteins				
**IRS2**	1.3	0.87	0.68	
PBXIP1	1.2	0.83	0.42	(35)
**DCBLD1**	1.2	0.86	0.56	
**ULK1**	1.2	0.85	0.58	
**NAA38**	1.2	0.82	0.44	
**LRP10**	1.2	0.82	0.49	
**CEP120**	1.2	0.84	0.42	
DIAPH3*	1.2	0.84	0.56	(36)
**ANKRD11**	1.2	0.86	0.53	
KEN-box containing proteins				
TK1	3.6	0.97	0.53	(37)
TACC3	1.9	0.94/0.90	0.54/0.49	(38)
TOP2A	1.8	0.86	0.44	(39)
**MKI67**	1.5	0.89/0.86	0.45/0.48	
CUEDC2	1.4	0.99	0.71	(40)
**GPBP1**	1.3	0.81	0.61	
**UHRF2**	1.2	0.88	0.61	
KIF23	1.2	0.87	0.45	(41)
**PNPLA8**	1.2	0.92	0.61	
**KDM2A**	1.2	0.94	0.66	
**PRPF38B**	1.2	0.92	0.44	
DLGAP5	1.2	0.89	0.6	(42)
D- and KEN-box containing proteins				
CKAP2	3.2	D: 0.95 K: 0.81	D: 0.59 K: 0.48	(43)
KIF11	2.4	D: 0.95 K: 0.81	D: 0.62 K: 0.49	(44)
GMNN**	2.2	D: 0.97 K: 0.84	D: 0.57 K: 0.52	(45)
**KDM3A**	1.2	D: 0.80 K: 0.81	D: 0.61 K: 0.66	
BUB1B	1.2	D: 0.92/0.85 K: 0.83/0.86	D: 0.47/0.64 K: 0.47/0.49	(46)

##### IRS2 Levels Are Controlled by Cdh1 in a Proteasome-Dependent Manner

**Fig. 2:**
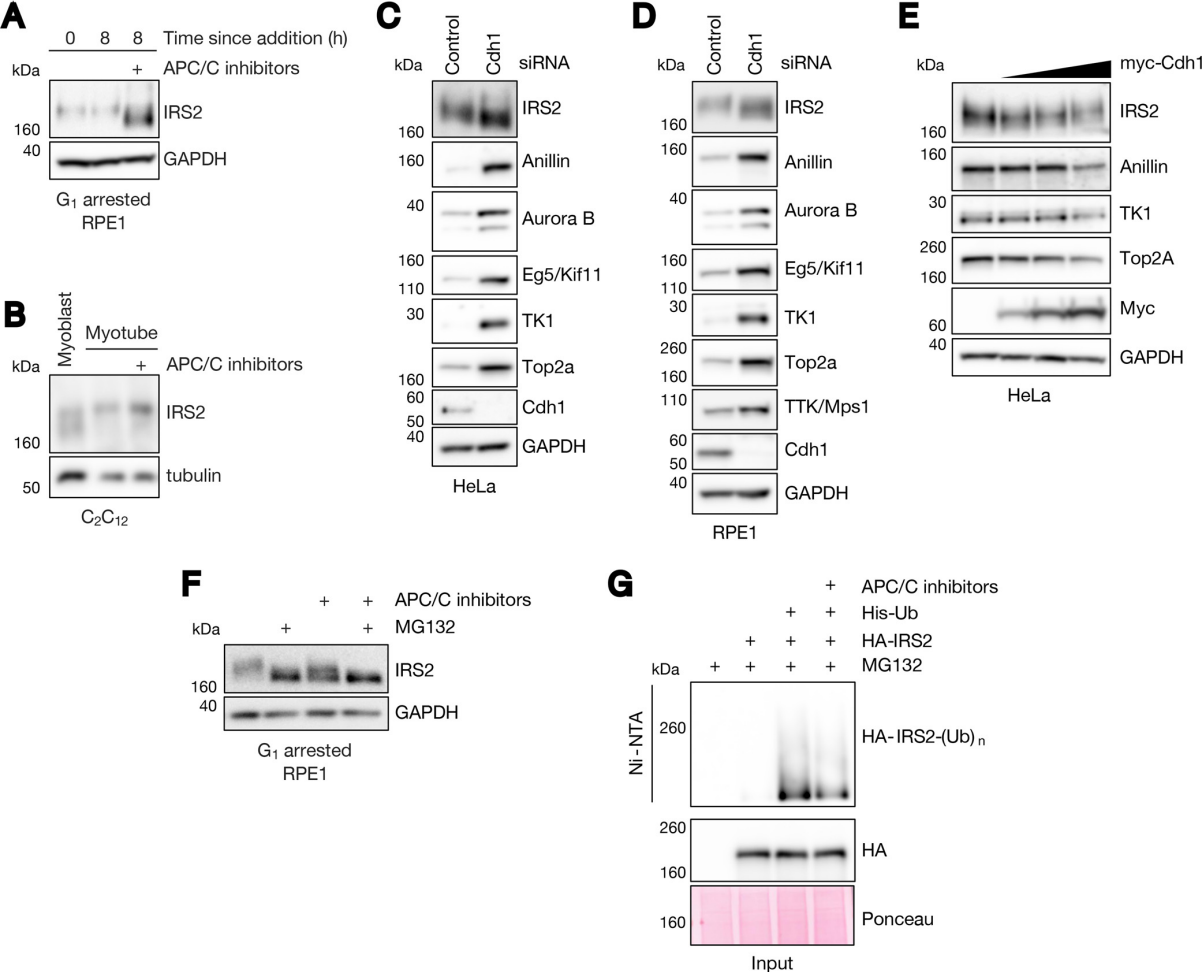
**IRS2 levels are controlled by Cdh1 in a proteasome-dependent manner.***A*, Cells were treated identically to what is described in Fig. 1*A*, and IRS2 abundance was measured by immunoblot. *B*, C_2_C_12_ myoblasts were induced to differentiate through serum withdrawal and supplementation with insulin, transferrin, and selenium (ITS). After 3 days of differentiation, myotubes were acutely treated with either DMSO or APC/C inhibitors. After eight hours of drug treatment, myotubes were collected and IRS2 levels from all samples were analyzed by immunoblotting. *C*–*D*, Asynchronous HeLa (*C*) and RPE1 (*D*) cells were transfected with either a control or Cdh1-directed siRNA for 24 h. Cells were allowed to grow for an additional 24 h before collection and analysis of the indicated protein levels in lysate by immunoblot. *E*, HeLa cells were mock transfected or transfected with increasing amounts of a plasmid encoding myc-tagged human Cdh1 for 24 h. Cells could grow for an additional 24 h before collection and analysis of the indicated protein levels by immunoblot. *F*, RPE1 cells were arrested in G_1_ with 1 μm palbociclib for 20 h. Following G_1_ arrest, cells were treated with DMSO, APC/C inhibitors, MG132, or a combination of APC/C inhibitors and MG132 for an additional 8 h. Cells were harvested, and lysates were analyzed by immunoblot for IRS2 abundance. *G*, 6xHis-tagged ubiquitin conjugates were isolated from HeLa cell lysates using Ni-NTA agarose resin. Lysates were derived from cells expressing 6xHis-ubiquitin and HA-tagged IRS2 that were treated with MG132 alone or in combination with APC/C inhibitors. Resin eluate and inputs were probed by immunoblot using an HA antibody, and Ponceau staining was used as a loading control.

Examining our list of 26 putative substrates, we focused our attention on IRS2—one of two major adaptors that promotes signaling through the insulin and insulin-like growth factor 1 receptors (IR/IGF1R). Using conditions identical to those under which the proteomics experiment was conducted, we validated that IRS2 was up regulated at the protein level under APC/C inhibition in G_1_-arrested RPE1 cells by immunoblot (Fig. 2*A*). Seeking to further validate this result in a distinct physiological context wherein APC/C^Cdh1^ activity is maintained in the absence of a pharmacological G_1_ blockade (47), we asked whether APC/C inhibition in terminally differentiated C_2_C_12_ myotubes also increases IRS2 protein abundance. C_2_C_12_ myoblasts easily differentiate into multinucleated myotubes following serum withdrawal and supplementation with growth factors (supplemental Figs. S3A–S3B). To validate that the APC/C is active in this system, we transfected C_2_C_12_ myoblasts with a model APC/C substrate (N-terminal fragment of cyclin B1 fused to EGFP; NT-CycB-GFP), allowed cells to differentiate into myotubes, and found that APC/C inhibition stabilized NT-CycB-GFP (supplemental Fig. S3C). Similarly, we found that acute APC/C inhibition in myotubes also resulted in an accumulation of IRS2 protein (Fig. 2*B*), thereby validating this finding from our G_1_ experiment in RPE1 cells in an independent system.

To exclude the possibility that the change in IRS2 abundance that we observed following APC/C inhibition was because of off-target effects of the small molecule APC/C inhibitors, we depleted Cdh1 using RNAi to block APC/C^Cdh1^ activity in HeLa, RPE1, and asynchronous C_2_C_12_ cells (Fig. 2*C*–2*D*, supplemental Fig. S3D). As expected, we found that Cdh1 knockdown caused an accumulation of endogenous IRS2 as well as several other previously reported APC/C substrates compared with control-transfected cells (Fig. 2*C*–2*D*).

To address whether increased APC/C^Cdh1^ activity is sufficient to reduce IRS2 levels, we overexpressed myc-tagged human Cdh1 in HeLa cells. We found that, when expressed at sufficiently high levels, Cdh1 reduced the levels of both IRS2 as well as other known APC/C^Cdh1^ substrates including anillin, TK1, and Top2a (Fig. 2*E*) (37, 39, 48). The requirement that Cdh1 be expressed at high levels to observe this effect is likely because of Cdk-dependent inhibitory phosphorylation of Cdh1 limiting its ability to activate APC/C under sub-saturating conditions (49).

We next sought to confirm that the increase in IRS2 protein observed under APC/C inhibition was because of impaired targeting of IRS2 to the proteasome. To test this, we arrested RPE1 cells in G_1_ using palbociclib and acutely treated them with APC/C inhibitors and/or a proteasome inhibitor (MG132) for 8 h. This experiment revealed that APC/C inhibition or proteasome inhibition each resulted in an accumulation of IRS2 (Fig. 2*F*). Notably, co-inhibition of the APC/C and the proteasome did not result in additional stabilization of IRS2, indicating that the increase in IRS2 we observed under APC/C inhibition was solely a consequence of its impaired proteasomal degradation. Consistent with this observation, we found that APC/C inhibition decreased the polyubiquitylation of HA-tagged IRS2 in HeLa cells treated with MG132 (Fig. 2*G*).

##### IRS2 Levels and Phosphorylation Fluctuate in a Cell-Cycle Dependent Manner

**Fig. 3:**
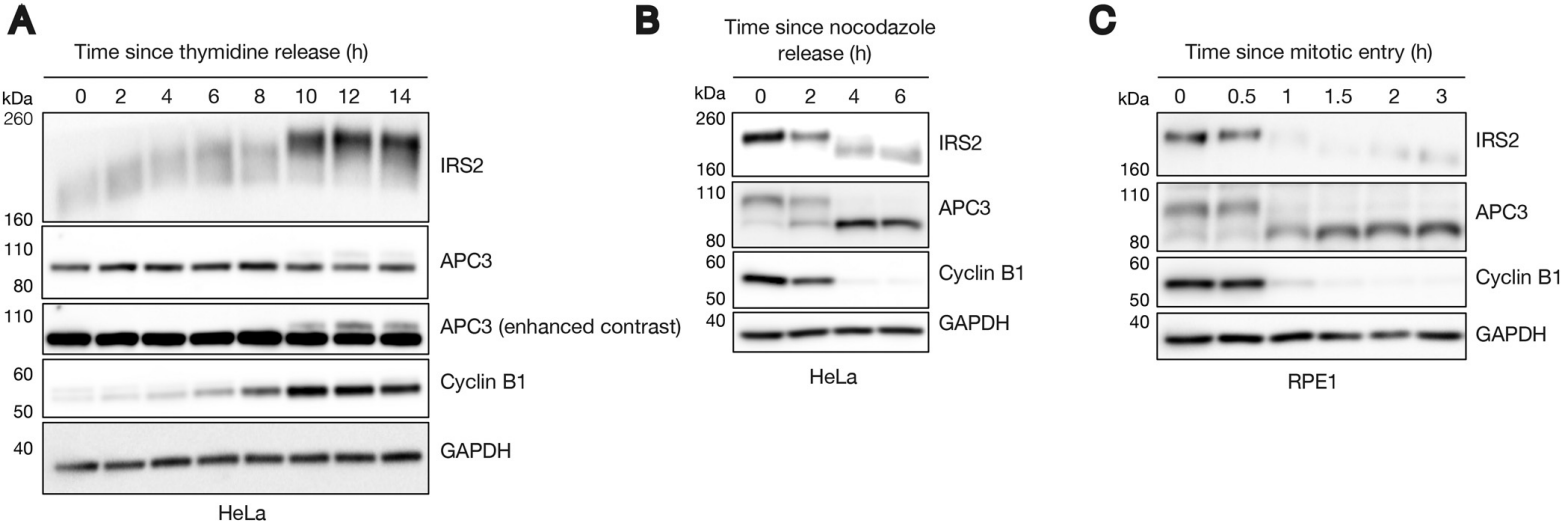
**IRS2 levels and phosphorylation fluctuate in a cell-cycle dependent manner.***A*, HeLa cells were synchronized by double thymidine block and released into S-phase in the presence of nocodazole. Lysates were harvested and analyzed by immunoblotting for IRS2 and cell cycle markers. *B*, HeLa cells were synchronized by single thymidine-nocodazole block and released into prometaphase. Mitotic cells were collected by mitotic shake-off and re-plated. Time points were taken every two hours as cells exited M-phase. Lysates were harvested and analyzed by immunoblotting for IRS2 and cell cycle markers. *C*, RPE1 cells were synchronized in G_2_ by treatment with the Cdk1 inhibitor RO3306. After 18 h, cells were switched to fresh media and were allowed to enter mitosis (∼35 min following drug removal). At mitotic entry, cells were collected by mitotic shake-off and were re-plated (0 h). Time points were taken as cells exited M-phase and entered G_1_. Lysates were harvested and analyzed by immunoblotting for IRS2 and cell cycle markers.

To test whether IRS2 levels fluctuate during the cell cycle as expected for an APC/C substrate (*i.e.* peaking during M-phase and falling rapidly at mitotic exit), we synchronized HeLa cells in early S-phase by double thymidine block and tracked IRS2 protein abundance leading into mitotic entry by immunoblot (Fig. 3*A*). As is typical for APC/C substrates, IRS2 levels correlated with cyclin B1 abundance. Furthermore, IRS2 levels positively correlated with the decreased electrophoretic mobility of APC3, which is caused by its extensive mitotic phosphorylation. To assess IRS2 levels at mitotic exit, we thymidine-nocodazole synchronized HeLa cells, released them into prometaphase, and tracked IRS2's abundance through mitotic exit (Fig. 3*B*). Again, IRS2 protein abundance correlated with cyclin B1 levels and APC3 phosphorylation. The same behavior was observed in RPE1 cells that were synchronized and released from late G_2_ by treatment with the Cdk1 inhibitor RO3306 and tracked over the course of progression through M-phase and into G_1_ (Fig. 3*C*). Based on these data, we conclude that IRS2 protein levels fluctuate in a cell cycle-dependent manner that is consistent with other known APC/C substrates. These data are also consistent with IRS2 being a potential APC/C^Cdc20^ substrate.

Our cell cycle analysis experiments revealed that IRS2 is hyperphosphorylated during mitosis as indicated by a marked electrophoretic mobility shift. This supports previous reports that IRS2 is phosphorylated by Plk1 during mitosis (50). Furthermore, this behavior may also require Cdk1 activity given that HeLa cells released from a double thymidine block into Cdk1 inhibitor RO3306 did not display an observable shift in IRS2 mobility as compared with those released into control (DMSO) treatment (supplemental Fig. S4). IRS2 abundance still peaked normally at this time point in the presence of RO3306, suggesting that the increase in IRS2 abundance was not dependent on Cdk1 activity. Together, these results support previous findings (50) that IRS2 is subject to cell-cycle dependent phosphorylation and demonstrate that its abundance peaks in M-phase and falls in early G_1_ in multiple cell lines.

##### Cdh1 Control of IRS2 Degradation Depends on an IRS2 d-Box Motif

**Fig. 4:**
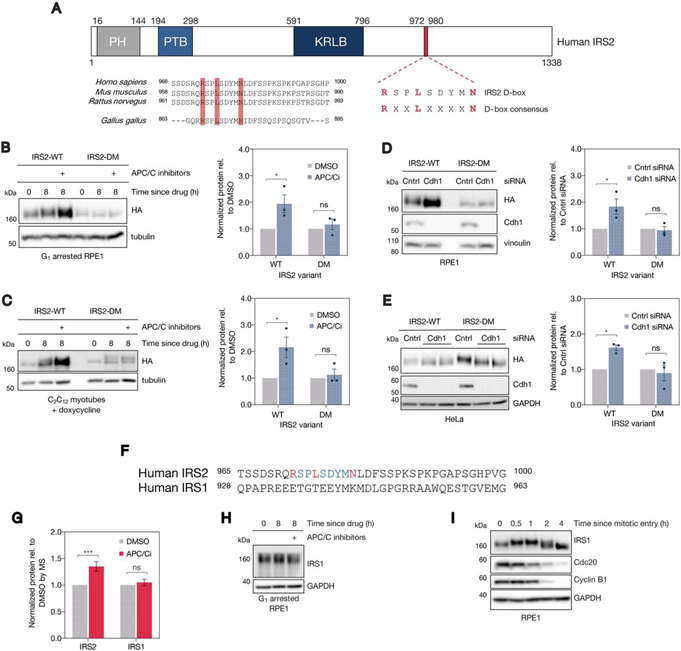
**Cdh1's ability to control IRS2 levels depends on a C-terminal d-box motif.***A*, (*top*) Schematic depicting IRS2's protein domain structure. PH = pleckstrin homology domain, PTB = phosphotyrosine binding domain, KRLB = kinase regulatory-loop binding region. IRS2's C-terminal full d-box motif is highlighted in red. (*bottom*) Comparison of IRS2's D-box conservation among amniotes. *B*, RPE1 cells stably expressing lentivirus-derived, doxycycline-inducible, C-terminally HA-tagged IRS2 constructs were arrested in G_1_ with palbociclib for 20 h. Following arrest, samples were either collected or DMSO or APC/C inhibitors were added for an additional 8 h. Quantification of immunoblots shown at right: HA levels were normalized to a loading control and are plotted relative to DMSO levels. Error bars = mean ± S.E. *: *p* = 0.0187; ns: *p* = 0.816. *C*, C_2_C_12_ myoblasts stably expressing lentivirus-derived, doxycycline-inducible, C-terminally HA-tagged IRS2 constructs were grown to confluence and switched to low serum media supplemented with ITS (differentiation media) and doxycycline. Cells were allowed to differentiate into myotubes for 3 days (with media refreshment every 24 h), at which point (0 h) either DMSO or APC/C inhibitors for an additional 8 h in the presence of doxycycline. Quantification of immunoblots shown at right: HA levels were normalized to a loading control and are plotted relative to DMSO levels. Error bars = mean ± S.E. *: *p* = 0.0118; ns: *p* = 0.910. *D*, Asynchronous RPE1 cells stably expressing lentivirus-derived, doxycycline-inducible C-terminally HA-tagged IRS2 constructs were transfected with a nontargeting (control) siRNA or an siRNA directed against Cdh1 for 24 h in the absence of doxycycline. Quantification of immunoblots shown at right: HA levels were normalized to a loading control and are plotted relative to DMSO levels. Error bars = mean ± S.E. *: *p* = 0.0132; ns: *p* = 0.963. *E*, Asynchronous HeLa cells stably expressing lentivirus-derived, N-terminally FLAG-HA tagged IRS2 constructs were transfected with a nontargeting (control) siRNA or an siRNA directed against Cdh1 for 24 h. Quantification of immunoblots shown at right: HA levels were normalized to a loading control and are plotted relative to DMSO levels. Error bars = mean ± S.E. *: *p* = 0.0131; ns: *p* = 0.803. *F*, Comparison of the Hs IRS2 D-box sequence with the corresponding region from Hs IRS1. *G*, MS-quantified IRS1 and IRS2 abundance in G_1_ APC inhibitor proteomics (Fig. 1). IRS1 abundance was quantified based on 5 peptides (4 unique) in 3 biological replicates; IRS2 was quantified based on 3 peptides (all unique) in 3 biological replicates. *H*, RPE1 cells were subject to the same conditions described in Fig. 1*A*, and cell lysates were analyzed by immunoblotting for IRS1 abundance *I*, RPE1 cells were treated as in Fig. 3*C*. Cell lysates were analyzed by immunoblotting for IRS1 abundance.

Using the SLiMSearch analysis tool (16), we found that IRS2 contains four minimal D-box motifs (RxxL), one extended D-box motif (RxxLxxxxN) and no KEN-box motifs. Of the four minimal D-box motifs, none bears strong consensus similarity to previously validated D-box motifs, and one exists in a highly structured region of the protein (34). Because of its high SLiMSearch parameter scores (Table I), we focused our efforts on determining whether the extended D-box motif located in the C-terminal third of IRS2 is required for its APC/C^Cdh1^-dependent stability. IRS2's extended D-box (amino acids 972–980 in human IRS2) is highly conserved in amniotes despite overall divergence in much of the C terminus (Fig. 4*A*), suggesting that this sequence likely has a conserved function.

To test whether IRS2's full D-box is relevant for its Cdh1-dependent degradation, we generated a mutant IRS2 construct bearing an R972A D-box mutation (DM), which was expected to abrogate its function as a D-box (51). To investigate the effect of expressing the IRS2-DM construct in cells, we generated doxycycline-inducible, C-terminally HA-tagged IRS2-WT and IRS2-DM RPE1 and C_2_C_12_ cell lines. In an effort to avoid saturating the system and overexpression artifacts, we sought to express tagged IRS2 variants at low levels relative to the endogenous protein (supplemental Fig. S5A). We were able to detect the tagged proteins in the RPE1 cells without adding doxycycline, so we avoided using it in this cell line to eliminate the possibility of observing off-target effects of doxycycline treatment. As a caveat of this approach, we observed that the basal expression level of IRS2-DM was about 2-fold lower than that of IRS2-WT (supplemental Fig. S5B). This was likely because of differences in lentiviral titer when the stable cell lines were generated. Still, we demonstrated that changes in transgene expression were quantifiable within the linear range for both cell lines (supplemental Fig. S5B). Because the relevant comparison is between controls (either DMSO or control siRNA) and APC/C inhibition (either APC/C inhibitors or Cdh1 siRNA) and not between IRS2-WT and IRS2-DM, we reasoned that these experiments were still interpretable. Using these cell lines, we found that APC/C inhibition following G_1_ arrest caused accumulation of IRS2-WT but not IRS2-DM (Fig. 4*B*). The degree of accumulation of the WT protein depended on the dose of APC/C inhibitors used (supplemental Fig. S5C).

In the C_2_C_12_ cells stably expressing C-terminally HA-tagged IRS2 variants, transgene expression was undetectable in differentiated myotubes in the absence of doxycycline (data not shown), so we selected a low dose that maintained close-to-endogenous expression levels in myoblasts (supplemental Fig. S5A). Cells were differentiated for 3 days in low-serum media. Following differentiation, myotubes were switched to new media containing fresh doxycycline and either DMSO or APC/C inhibitors (the “0 h” lane in Fig. 4*C*) and were collected 8 h later. Because of the doxycycline refreshment, myotubes exhibited a slight increase in transgene expression between 0 h (the time of drug addition) and 8 h (the time of collection). We observed a strong increase in levels of HA-tagged IRS2-WT in the presence of APC/C inhibitors compared with DMSO-treated myotubes. In contrast, levels of HA-tagged IRS2-DM remained constant both in the presence and absence of APC/C inhibitors (Fig. 4*C*).

To further validate the Cdh1-dependence of IRS2's D-box motif, we asked whether Cdh1 knockdown by siRNA could stabilize the IRS2-DM protein. Using asynchronous RPE1 cells stably expressing C-terminally HA-tagged IRS2-WT and IRS2-DM, we found that Cdh1 knockdown by siRNA caused an accumulation of IRS2-WT relative to control-transfected cells but not IRS2-DM (Fig. 4*D*). The same result was obtained in HeLa cells stably expressing N-terminally FLAG-HA-tagged IRS2-WT and IRS2-DM constructs subject to the same conditions (Fig. 4*E*).

IRS1 (the other primary adaptor protein for IGF1R and IR) shares 75% sequence homology with IRS2's N terminus and 35% homology with its C terminus (52) but does not share the D-box motif found in IRS2's C terminus (Fig. 4*F*). In keeping with our hypothesis that Cdh1-mediated control of IRS2 is D-box dependent, IRS1 levels did not increase in G_1_-arrested RPE1 cells treated with APC/C inhibitors as measured by either MS (Fig. 4*G*) or immunoblot (Fig. 4*H*). Furthermore, although it did display a change in electrophoretic mobility compatible with mitotic phosphorylation, unlike IRS2, it did not decrease in abundance at mitotic exit in RPE1 cells (Fig. 4*I*). Taken together, the findings described above indicate that APC/C^Cdh1^ controls IRS2 levels in manner that depends on its C-terminal D-box motif.

##### IRS2 is Required for Normal Expression of Many Proteins Involved in Mitosis

Many reported APC/C^Cdh1^ substrates (including several of those identified in our initial proteomics screen) are required for normal cell cycle progression. Because IR/IGF1R transduction promotes a variety of transcriptional programs (2), we hypothesized that IRS2 might promote the expression of proteins involved in cell cycle control. To investigate this, we generated two IRS2 knockout RPE1 cell lines using CRISPR/Cas9 (Fig. 5*A*), henceforth referred to as ΔIRS2-A and ΔIRS2-B. Using these cells, we again employed TMT-coupled quantitative proteomics. The proteomes of WT, ΔIRS2-A, and ΔIRS2-B cell lines were analyzed in biological triplicate, and relative abundances were ascertained based on TMT reporter ion signal-to-noise values. Unsurprisingly, we did not detect peptides from IRS2 in this experiment. This is likely because IRS2 was absent from six of the nine TMT channels, which lowered its overall abundance in the pool of samples analyzed and therefore lowered its chances of being detected. Hierarchical clustering indicated that the proteomes of the two knockout cell lines analyzed were more similar to each other than either knockout cell line was to WT (supplemental Fig. S6A), suggesting that deletion of IRS2 produced similar effects in both cell lines. To exclude aberrancies that may have accrued during the CRISPR process or as a result of clonal expansion, we focused the scope of our analysis to proteins that changed significantly (*p* < 0.05) by more than 20% in both IRS2 knockout clones relative to the WT cell line (Figs. 5*B*–5*C*). We found 239 proteins that decreased by >20% in both IRS2 knockout lines relative to the WT line and 300 proteins that increased by >20% (Figs. 5*B*–5*C*, supplemental Fig. S6B).

We conducted gene enrichment analysis of the proteins that increased (supplemental Fig. S6C–S6D) or decreased (Fig. 5*D*) by >20% in both knockout cell lines relative to WT cells. Of the 239 proteins that were depleted by >20% in both knockout cell lines, we found a statistical over-representation of proteins participating in metabolic processes characteristic of IR signal transduction. Notably, we also found an over-representation of proteins involved in mitotic cell cycle regulation in this subset (Fig. 5*D*). This suite of proteins included regulators of mitotic entry and exit as well as several factors involved in spindle assembly (Fig. 5*E*). Importantly, IRS2 knockout cells divide as the same rate as WT cells (supplemental Fig. S7A–S7B), indicating that this down regulation is not because of a bulk loss of viability or cell cycle arrest. Consistent with the fact that strong depletion of most critical cell cycle regulators renders cells inviable, most of the observed changes in cell cycle-related genes were relatively modest (supplemental Fig. S7C). Based on these data, we conclude that IRS2 is important for promoting the expression of a suite of proteins involved in orchestrating the mitotic cell cycle, and deletion of IRS2 stunts their expression in RPE1 cells.

##### IRS2 Expression Promotes a Functional Spindle Assembly Checkpoint

**Fig. 6:**
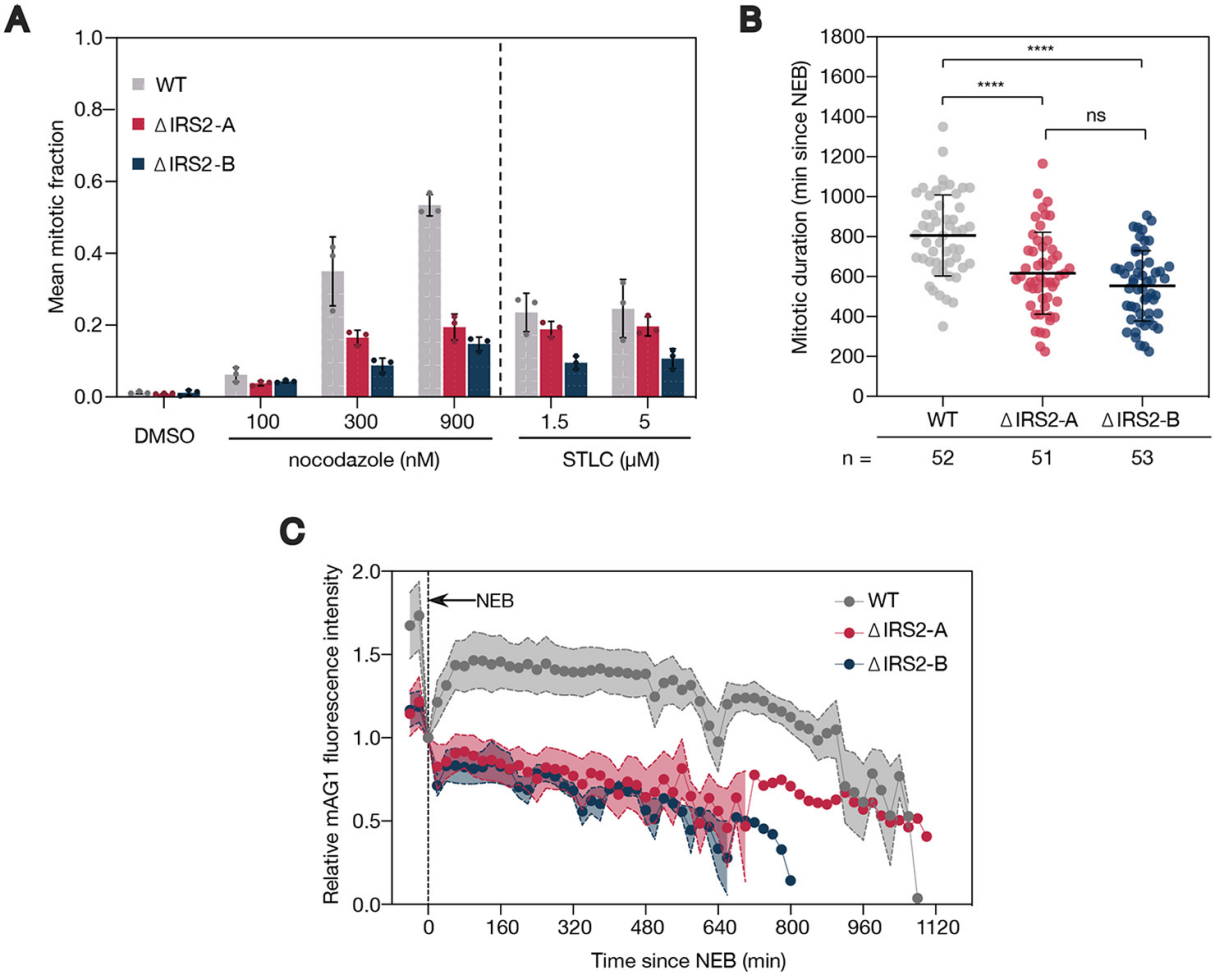
**IRS2 expression promotes a functional spindle assembly checkpoint.***A*, Analysis of fraction of cells in mitosis for RPE1 WT and IRS2 knockout cell lines treated with the indicated doses of nocodazole and S-trityl-l-cysteine (STLC) for 18 h. Mitotic fraction measurements were made using a high content fixed cell imaging assay based on DAPI intensity of stained nuclei. Error bars = mean ± S.D. *B*, Asynchronous RPE1 WT or IRS2 knockout cell lines were treated with 300 nm nocodazole and imaged every 5 mins by widefield time lapse microscopy for 36 h. Each point represents an individual cell's mitotic duration, measured as the time from nuclear envelope breakdown (NEB) to division, slippage, or cell death. Error bars = mean± S.D. *p*-values were calculated by one-way ANOVA. **** = *p* < 0.0001. ns = not statistically significant. C, Asynchronous RPE1 WT or IRS2 knockout cell lines expressing mAG1-geminin(1-110) were treated as in (*C*). mAG1 fluorescence intensity was measured from nuclear envelope breakdown (NEB) until division, slippage, or cell death (*n* = 10 for all three cell lines). Error bars = mean ± S.E. Fluorescence intensity was background subtracted and normalized to intensity at NEB.

Because many of the factors that were depleted in IRS2 knockout cell lines are involved in regulating the events of mitosis, we sought to investigate whether IRS2 knockout cell lines display phenotypic differences from WT cells under conditions of mitotic stress—in this case, activation of the spindle assembly checkpoint (SAC). Using a high content nuclear imaging assay to measure mitotic fraction (17), we asked whether IRS2 knockout cell lines display mitotic arrest differences compared with WT cells when treated with spindle poisons in a fixed cell assay. Wild type cells treated with nocodazole (a microtubule destabilizing agent) arrested in mitosis in a dose-dependent manner, whereas both IRS2 knockout cell lines displayed depressed mitotic arrest (Fig. 6*A* and supplemental Fig. S8A). This was also true to a lesser extent in the presence of S-trityl-l-cysteine (STLC), an Eg5 inhibitor (Fig. 6*A*). Importantly, this was not because of a reduced rate of mitotic entry (supplemental Fig. S7B).

We further evaluated this phenotype by live-cell imaging. Consistent with the results from the fixed-cell assay, IRS2 knockout cell lines also had a significantly shorter mitotic duration compared with WT cells (*p* < 0.0001 in both cases) when treated with 300 nm nocodazole (Fig. 6*B* and supplemental Fig. S8B). We next analyzed the effect of IRS2 knockout on APC/C activity in cells expressing mAG1-geminin (1-101), an APC/C^Cdc20^ substrate that is stabilized by the spindle assembly checkpoint (53). We found that WT cells displayed an accumulation of mAG1 fluorescence early in mitotic arrest before a gradual reduction because of leaky APC/C activity (54). In contrast, both IRS2 knockout cell lines display depressed mAG1 accumulation, followed by a more rapid loss of fluorescence signal, consistent with higher APC/C^Cdc20^ activity because of a weakened checkpoint (Fig. 6*C*). This phenotype, along with the shorter mitotic duration and lower mitotic fraction in the presence of spindle poisons, is consistent with cells bearing a defective mitotic spindle assembly checkpoint. Based on these data, we conclude that IRS2 expression promotes a functional spindle assembly checkpoint in RPE1 cells.

## DISCUSSION

We conducted an unbiased proteomic screen for APC/C substrates in G_1_ cells treated with small molecule APC/C inhibitors. By analyzing our data set for proteins that were stabilized under APC/C inhibition and also contained well-predicted degron motifs, we identified several novel putative and previously reported APC/C substrates. Although the majority of the previously reported APC/C^Cdh1^ substrates that were quantified in our G_1_ proteomic experiment were stabilized following APC/C inhibition, some remained unchanged. There are several possible explanations for this result. First, some substrates may be APC/C^Cdh1^-accessible only under conditions or in tissue types that were not met by the experimental parameters that we used. Second, some proteins (*e.g.* FBXW5, ZC3HC1) (55, 56) were proposed to be APC/C^Cdh1^ substrates based on results obtained in Cdh1 overexpression systems, indicating that APC/C^Cdh1^ activity may be sufficient but not necessary to control their levels. Lastly, incomplete APC/C inhibition under our experimental conditions may have led to the continued degradation of particularly high-affinity APC/C substrates.

Based on the results of our screen, we provide evidence that IRS2, a critical mediator of IR/IGF1R signaling, is a direct APC/C^Cdh1^ substrate. We demonstrate that IRS2 is stabilized by APC/C inhibition and Cdh1 knockdown in multiple cell types and that this depends on IRS2's C-terminal D-box motif. In contrast, we find that IRS1, a closely related IRS2 paralog that lacks a D-box, is not subject to regulation by the APC/C. Taken together, these results show that APC/C activity directly controls IRS2 levels in a D-box dependent manner.

We identified a high-mobility form of IRS2 that accumulates under APC/C inhibition, likely corresponding to a difference in phosphorylation given that IRS2 has ∼150 annotated threonine, serine, and tyrosine phosphorylation sites (57). This suggests that IRS2's APC/C-dependent stability could be regulated by phosphorylation, possibly at sites near or within the d-box. Consistent with this possibility, IRS2 phosphorylation is known to impact its stability in other contexts, including following prolonged exposure to insulin or following mTOR activation (2). Furthermore, there is a strong precedent for phospho-regulation of APC/C degrons modulating substrate stability under specific conditions (58, 59, 60). Understanding how phosphorylation of IRS2 influences its recognition by both Cdh1 and Cdc20 is an interesting question for future studies.

Many APC/C substrates are involved in cell cycle regulation, and previous studies have suggested a relationship between IRS2 and cell cycle progression. IRS2 can stimulate cell cycle entry via Cdk4 activation (61) and is important for sustaining proliferation in 32D myeloid cells and pancreatic β cells (62, 63). Based on these findings and our identification of IRS2 as an APC/C substrate, we further investigated the role of IRS2 in regulating cell division. Proteomic analyses of RPE1 cells lacking IRS2 reveal lower expression of well-characterized cell cycle proteins compared with WT cells. Because these proteins are involved in critical processes like cytokinesis, DNA replication, cell cycle transitions, and spindle assembly, we investigated whether IRS2 knockout cell lines display cell cycle progression defects. We find that cells lacking IRS2 have an impaired ability to arrest following spindle assembly checkpoint activation in M-phase, thereby implicating IRS2 in promoting a functional spindle assembly checkpoint.

Despite the well-established importance of sustained IRS2 levels in many tissue types, little is known about what factors regulate its turnover. Although several distinct ubiquitin ligases control IRS1 stability (Fbxw8, Cbl-b, Fbxo40, SOCS1/3, MG53, and others) (8, 9, 10, 11, 12), only SOCS1/3 have been implicated in the ubiquitin mediated proteolysis of IRS2 [11] until now. Thus, our work establishes APC/C^Cdh1^ as the first known ubiquitin ligase that targets IRS2 but not IRS1. Furthermore, our results suggest that APC/C^Cdh1^-mediated IRS2 degradation is relevant in broad biological contexts because we were able to demonstrate this mechanism of regulation in multiple cell lines.

Over the past several years, a number of connections between growth factor signaling and APC/C-mediated regulation have emerged. SKIL/SnoN, an APC/C substrate involved in TGFβ signaling, implicates APC/C activity in modulating the expression of TGFβ target genes (64). Another APC/C substrate, CUEDC2, controls the stability of the progesterone receptor (65). Regarding IR/IGF1R signaling, connections to APC/C-mediated regulation have been opaquer. Multiple reports have shown that Cdh1 interacts with PTEN, a phosphatase that antagonizes signal transduction through the IR pathway by dephosphorylating phosphoinositide-3,4,5-triphosphate (PIP_3_) (66, 67). Others have demonstrated that components of the mitotic checkpoint complex (which inhibit APC/C^Cdc20^) potentiate IR signaling via IR endocytosis (68, 69). Despite these links, there have been no reports of direct APC/C substrates that are involved in IR signaling until now.

**Fig. 7:**
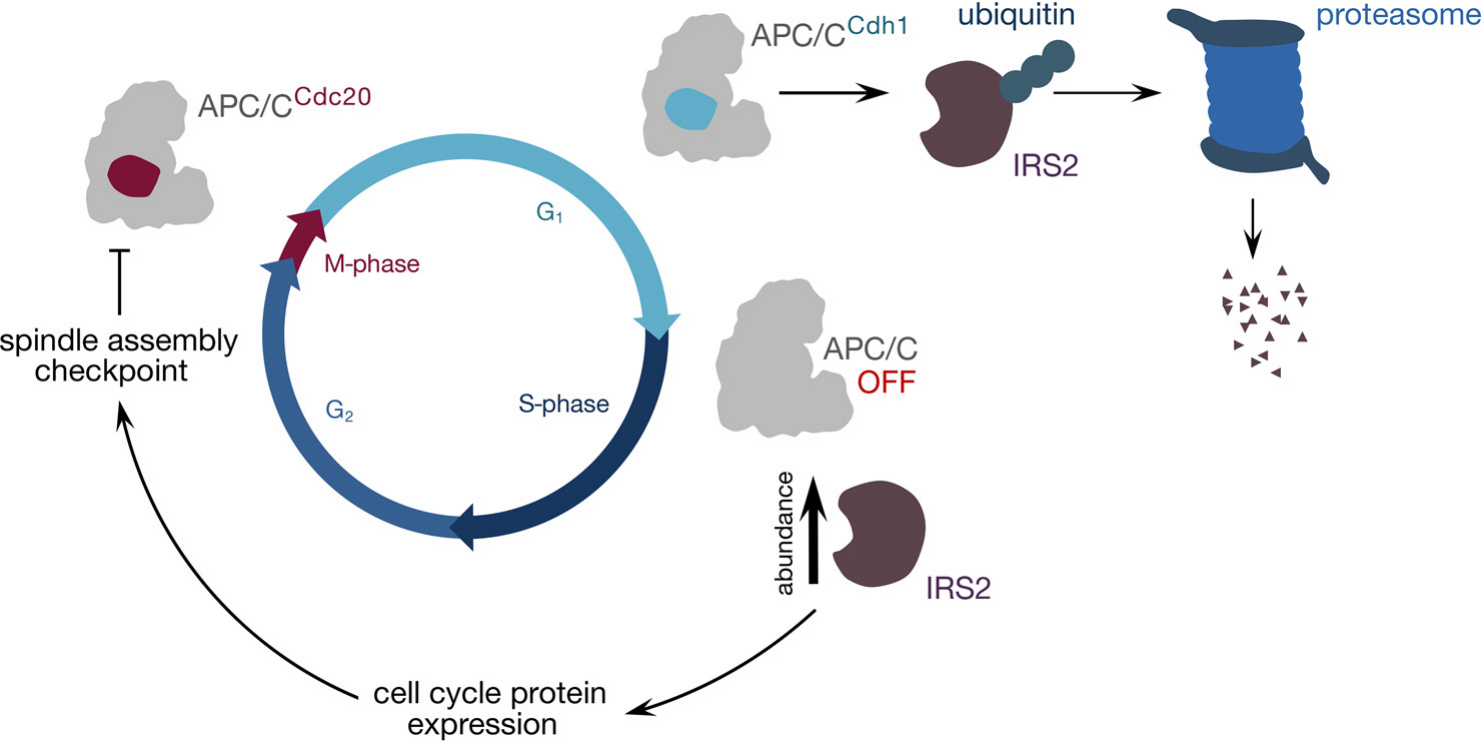
**Model for IRS2's role in cell cycle control.** IRS2 is targeted for proteasomal degradation by APC/C^Cdh1^ during G_1_. When APC/C is inactivated at the G_1_/S boundary, IRS2 protein accumulates, allowing it to stimulate the expression of cell cycle-related proteins either through IR-mediated action (71) or through another receptor tyrosine kinase. Some of the proteins that are regulated through this pathway may be required for a robust spindle assembly checkpoint, which directly inhibits APC/C^Cdc20^ during M-phase.

Based on the data presented here, we propose a model (Fig. 7) in which IRS2's APC/C-mediated degradation in G_1_ serves to limit IRS2-dependent signaling during G_1_. On APC/C inactivation, IRS2 is able to accumulate and stimulate signaling required for normal progression through later stages of the cell cycle, including the expression of proteins required for mitotic spindle checkpoint function. This model is consistent with previous studies that implicate IRS2 in promoting the expression of cell cycle-related genes, including mitotic cyclins (A and B) in mouse granulosa cells (70). Furthermore, IR signal transduction promotes the expression of Plk1 (a mitotic kinase) and CENP-A (a centromere protein) in β cells through a mechanism that appears to depend on IRS2 rather than IRS1 (63, 71). An important future experiment to address the physiological relevance of APC/C-mediated degradation of IRS2 will be to evaluate phenotypes in IRS2 D-box mutant mice.

Our findings suggest that APC/C^Cdh1^ modulates IRS2-dependent signaling but not IRS1-dependent pathways. In IRS2-deficient mice with consequent type 2 diabetes, some have attributed the reduced β cell mass to a failure of β cells to re-enter the cell cycle following division (63). Our findings that APC/C^Cdh1^ inhibition stabilizes IRS2 and that IRS2 promotes the expression of cell cycle regulatory proteins, coupled with data from others showing that IRS2 can stimulate cell cycle entry (61), suggest that APC/C^Cdh1^ inhibition may represent a possible approach for stimulating proliferation in quiescent β cells via the stabilization of IRS2.

## DATA AVAILABILITY

All MS raw files are available through the PRIDE partner repository (30) with the data set identifier PXD018329 and 10.6019/PXD018329. All other data are available in the associated supplemental data files. Further information and requests for resources and reagents should be directed to the Lead Contact, Randy King (randy_king@hms.harvard.edu).
