# Supplementary material for: The Insulin Receptor Adaptor IRS2 is an APC/C Substrate That Promotes Cell Cycle Protein Expression and a Robust Spindle Assembly Checkpoint
Source: Mol Cell Proteomics. 2020 Nov 25;19(9):1450–67. doi: 10.1074/mcp.RA120.002069 (PMC8143631; doi:10.1074/mcp.RA120.002069)
Supplement: Supplementary file 1 [file mmc1.zip › mmc1/159856_1_supp_540475_qb9qfn.docx]

**Supplemental Data Legends**

***Figure S1***: *Related to Figure 1*

1. Asynchronous RPE1-FUCCI cells were treated with 1 µM palbociclib and imaged by fluorescence time lapse microscopy for 20 hours. Frames at 0, 10, and 20 hours are shown.
2. From the experiment shown in Figure S1B, mAG1-geminin (1-110) intensity was quantified at 0 hours (time of drug addition) and 20 hours. Each point represents the maximum mAG1-geminin (1-110) intensity of an individual cell at the given time point. ns : not significant ; **** : *p*<0.0001.
3. Asynchronous RPE1-FUCCI cells were treated with 1 μM palbociclib for 20 hours. Following G_1_ arrest, cells were treated with either DMSO or APC/C inhibitors (6 µM proTAME + 50 µM apcin) and imaged by fluorescence widefield time lapse microscopy for an additional eight hours.
4. Quantification of the experiment shown in S1C, as explained in S1B. Error bars = SD among all of the cells quantified for each condition. ns: not significant ; **** : *p*<0.0001.
5. RPE1-FUCCI cells were treated with 1 μM palbociclib for 20 hours. Following G_1_ arrest, cells were either switched to fresh media containing no drug (“wash out”), fresh media containing the same concentration of palbociclib (“palbociclib”), or fresh media containing the same concentration of palbociclib and APC/C inhibitors (palbociclib + APC/Ci). mAG1-geminin (1-110) intensity was quantified for all cells in a given frame every 2.7 hours for 16 hours. Each data point represents the mean intensity ± SEM for at least 90 cells from two different frames for each condition.

**Figure S2**: *Related to G_1_ APC/C inhibitor proteomics (quality control data)*

1. Hierarchical clustering and correlation plot for the nine samples (three conditions, each in biological triplicate) analyzed in the G_1_ APC/C inhibitor proteomics experiment. Log_2_ ratios of each sample relative to the bridge channel were calculated and clustered by hierarchical clustering using Euclidean distance. Circle size and color represent Pearson correlations between the log_2_ ratios. Purple cluster = t_0_ (samples collected at the time of either DMSO or APC/C inhibitor addition); gray cluster = DMSO (8 hr); blue cluster = APC/C inhibitors (8 hr)
2. Coefficient of variation (CV) of protein quantification among biological replicates. Most proteins have a CV < 4%.
3. Boxplot for number of observations required for proteins with 15% change to obtain a significance level of 0.05 with a power of 0.95.
4. (*top*) TMT signal-to-noise ratios for the three peptides used to identify and quantify IRS2. Each bar represents the mean signal-to-noise ratio for each peptide ± SD across the three biological replicates analyzes. Individual data points for each replicate are shown. The identity of each peptide is shown in the table (*bottom*).

***Figure S3***: *Related to Figure 2B*

1. Asynchronous C_2_C_12_ myoblasts and 3-day differentiated C_2_C_12_ myotubes were lysed and MyoD levels were measured by immunoblotting.
2. Phase-contrast images of asynchronous (Day 0) C_2_C_12_ myoblasts and 3-day differentiated C_2_C_12_ myotubes.
3. Asynchronous C_2_C_12_ myoblasts were transfected with a plasmid coding for the N-terminal fragment of cyclin B1 (amino acids 1-88) fused to EGFP for 24 hours. Following transfection, cells were switched to low-serum differentiation media containing insulin, transferrin, and selenium (ITS) for three days with media refreshment every 24 hours. After 3 days, myotubes were acutely treated with either DMSO or APC/C inhibitors (6 µM proTAME + 50 µM apcin) for an additional 8 hours. Myotubes were then harvested, and lysates were analyzed for transgene expression by immunoblot.
4. Asynchronous C_2_C_12_ myoblasts were transfected with an siRNA directed against the mouse Cdh1 sequence for 18 hours before being maintained in fresh media for an additional 18 hours. Cells were harvested, and lysates were analyzed for the expression of the indicated proteins.

***Figure S4***: *Related to Figure 3*

HeLa cells were synchronized by double thymidine block and released into S-phase either in the presence of DMSO or 5 µM RO3306. Cells were harvested at the indicated time points for analysis of the given protein abundances by immunoblot.

***Figure S5***: *Related to Figure 4*

1. (*top*) Asynchronous RPE1 cells expressing doxycycline-inducible, C-terminally HA tagged IRS2 variants were treated with a dose range of doxycycline, and HA and IRS2 expression levels were analyzed by immunoblotting cell lysates with anti-HA and anti-IRS2 antibodies. Red = doxycycline dose used in **Figure 4B** and **Figure 4D**. (*bottom*) Asynchronous C_2_C_12_ cells expressing doxycycline-inducible, C-terminally HA tagged IRS2 variants were treated with a dose range of doxycycline. HA and IRS2 expression levels were analyzed by immunoblotting cell lysates with anti-HA and anti-IRS2 antibodies. Red = doxycycline dose used in **Figure 4C**.
2. (*left*) Asynchronous RPE1 cells expressing doxycycline-inducible, C-terminally HA-tagged variants were lysed, and a dilution series of lysate was analyzed for by immunoblot with an anti-HA antibody. The protein amount highlighted in red, 15 μg, was the amount loaded in **Figure 4B** and **Figure 4D**. The corresponding Coomassie stained gel is shown below. (*right*) Quantification of the experiment at left. Linear regression calculations were made, and lines were plotted over data. For WT, R^2^= 0.97. For DM, R^2^=0.99
3. *(left*) RPE1 cells stably expressing lentivirus-generated, C-terminally HA-tagged IRS2 wild type (WT) and R972A (DM) constructs were arrested in G_1_ with 1 µM palbociclib for 20 hours. Cells were then treated with either DMSO or the indicated dose range of APC/C inhibitors for an additional 8 hours. Cells were then harvested, and lysate was analyzed by immunoblot with an anti-HA antibody. The lane denoted t_0_ indicates a sample that was collected at the time of drug addition. (*right*) Quantification of the experiment at left. Plot shows HA intensity normalized to a loading control (either GAPDH or Ponceau) and to the DMSO condition. Error bars = mean ± SEM.

***Figure S6***: *Related to Figure 5*

1. Hierarchical clustering for the nine conditions analyzed by TMT-coupled quantitative mass spectrometry in wild type and ∆IRS2 cell lines.
2. Venn diagrams depicting proteins that (*top*) decrease significantly >20% relative to WT cells in both ∆IRS2 cell lines and (*bottom*) increase significantly >20% relative to WT in both ∆IRS2 cell lines.
3. Gene ontology (GO) term enrichment of proteins that increase in both ∆IRS2 cell lines relative to WT cells.
4. Heat map for proteins in the “oxidation-reduction process” GO category increasing in both ∆IRS2 cell lines relative to WT cells.

***Figure S7***: *Related to Figure 5*

1. Growth curves for IRS2 knockout cell lines.
2. Asynchronous RPE1 WT and IRS2 KO cells were imaged every five minutes by widefield time lapse microscopy for 36 hours. The fraction of cells that entered mitosis over this time span was measured manually, and cumulative frequency curves for mitotic entry were plotted. WT: n= 33 ; ∆IRS2-A: n=42; ∆IRS2-B: n=42.
3. Fractional abundance of cell-cycle related proteins shown in **Figure 5D-5E** depleted in ∆IRS2 cell lines relative to WT cells. ∆IRS2-A median abundance = 0.70 ; ∆IRS2-B median abundance = 0.66.

***Figure S8***: *Related to Figure 6*

1. Representative frames from high-content nuclear imaging experiment for mitotic fraction based on DAPI intensity. Asynchronous RPE1 WT or IRS2 KO cell lines were treated with 900 nM nocodazole for 18 hours before fixing and DAPI staining.
2. Asynchronous RPE1 WT and IRS2 KO cell lines were imaged every five minutes by widefield time lapse microscopy for 36 hours. Each point represents an individual cell’s mitotic duration, measured as the time from nuclear envelope breakdown (NEB) to division, slippage, or cell death. Error bars = mean ± SD. *p*-values were calculated using one-way ANOVA. ns = not statistically significant.
3. Asynchronous RPE1 WT and IRS2 KO cell lines were imaged as in (B). Images are representative of an unperturbed mitosis from NEB to cytokinesis for each cell line. Time (in minutes) in upper right corner indicates time since NEB.

***Figure S9***: *Related to Figures 1-2*

Extended immunoblots from Figures 1 and 2

***Figure S10***: *Related to Figures 3*

Extended immunoblots from Figure 3

***Figure S11****: Related to Figures 4-5*

Extended immunoblots from Figures 4 and 5

***Table S1***: APC/C inhibition in G_1_ proteomics

***Table S2***: Reported APC/C substrates identified by proteomics

***Table S3***: 204 protein subset

***Table S4***: APC/C inhibition in G_1_ SEQUEST search data

***Table S5***: IRS2 knockout cell proteomics

***Table S6***: IRS2 knockout SEQUEST search data

**Supplemental Video 1 (SV1)**: Time lapse video of RPE1 FUCCI cells arrested in palbociclib for 20 hours treated with DMSO. Cells were arrested with palbociclib for 20 hours, treated with DMSO, and imaged immediately every 8 minutes for an additional 19 hours.

**Supplemental Video 2 (SV2)**: Time lapse video of RPE1 FUCCI cells arrested in palbociclib for 20 hours treated with APC/C inhibitors. Cells were arrested with palbociclib for 20 hours, treated with APC/C inhibitors, and imaged immediately every 8 minutes for an additional 19 hours.

**Supplemental Video 3 (SV3)**: Time lapse video of RPE1 FUCCI cells arrested with palbociclib for 20 hours and then released into the cell cycle. Cells were arrested with palbociclib for 20 hours, followed by drug removal and switch to fresh media. Cells were imaged immediately following drug wash-out every 8 minutes for an additional 19 hours.
