# Supplementary material for: The Insulin Receptor Adaptor IRS2 is an APC/C Substrate That Promotes Cell Cycle Protein Expression and a Robust Spindle Assembly Checkpoint
Source: Mol Cell Proteomics. 2020 Nov 25;19(9):1450–67. doi: 10.1074/mcp.RA120.002069 (PMC8143631; doi:10.1074/mcp.RA120.002069)

Supplemental Figure 1

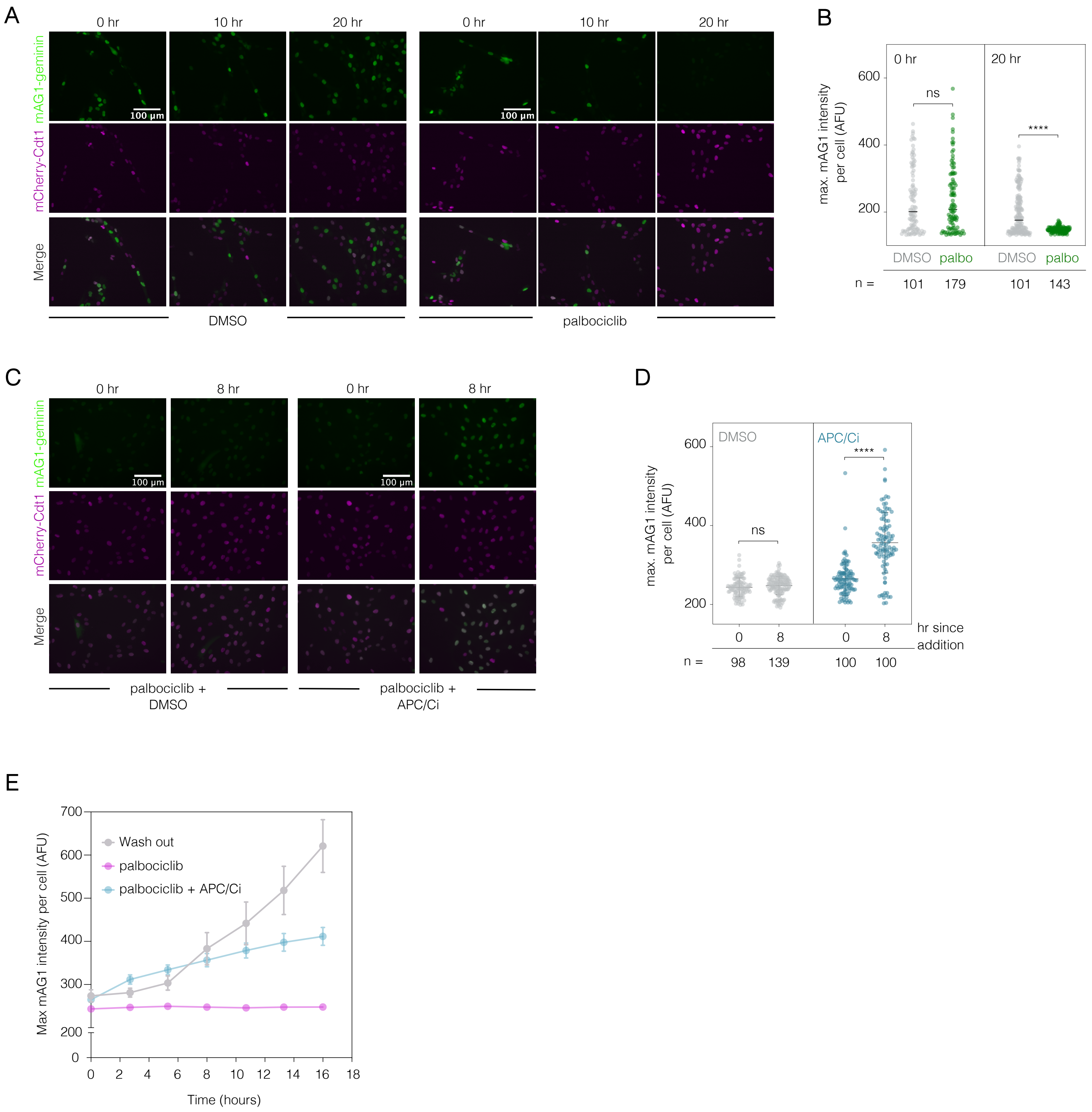

Supplemental Figure 2

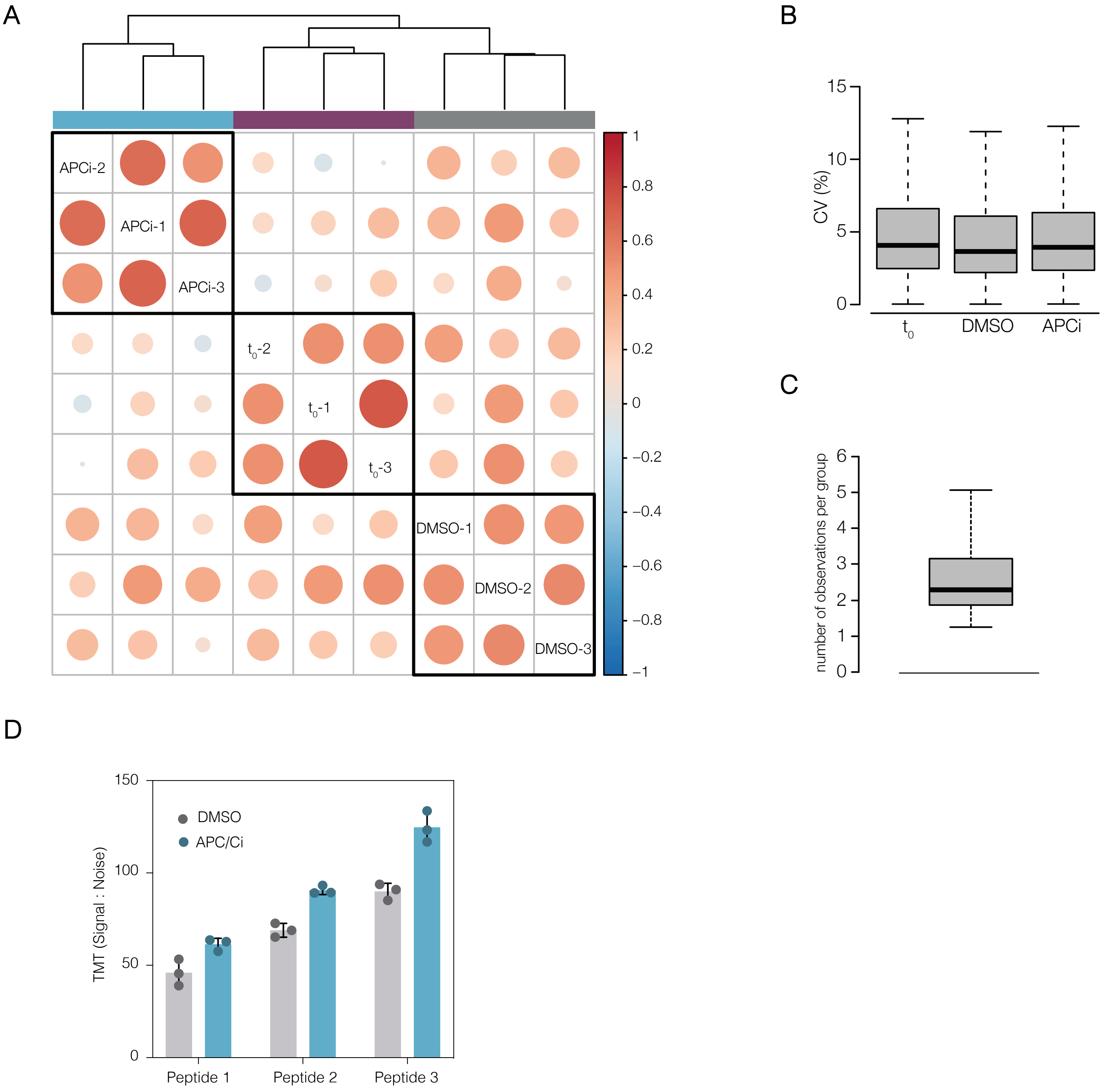

Peptide	Sequence
1	K.APYTCGGDSDQYVLMSSPVGR.I
2	K.VALLPAGGALQHSR.S
3	AAAAAAAAVPSAGPAGPAPTSAAGR.T

Supplemental Figure 3

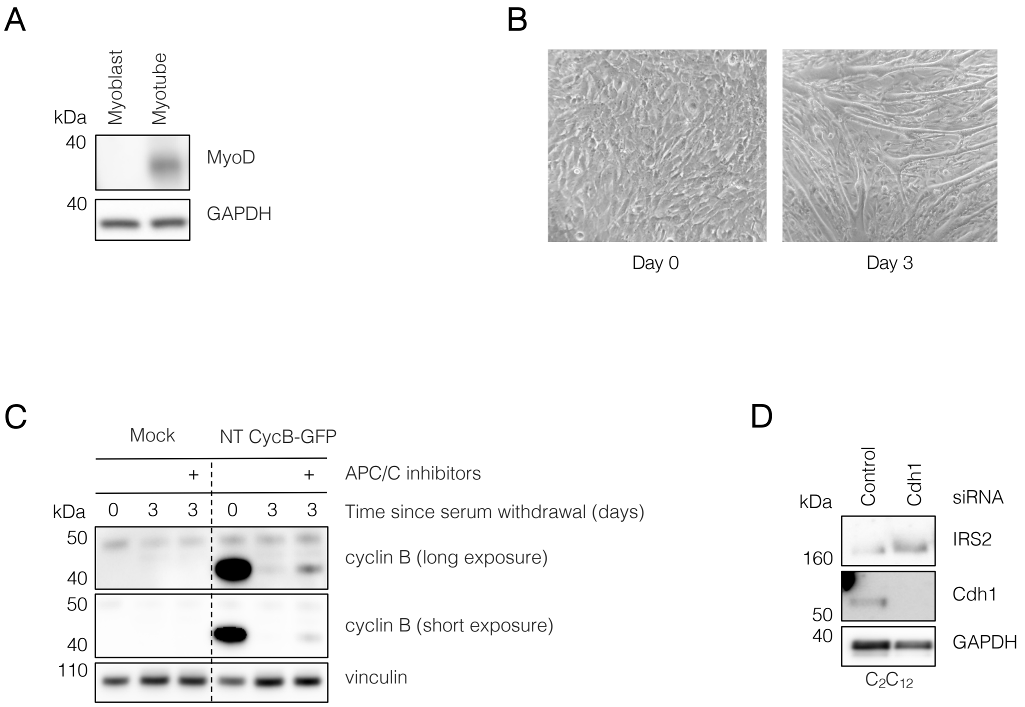

Supplemental Figure 4

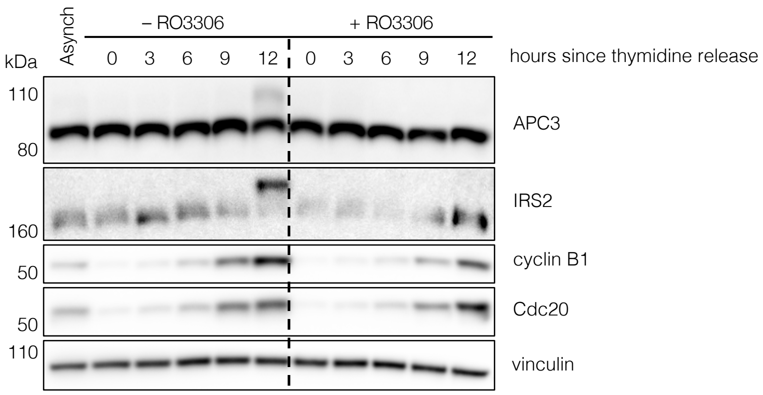

Supplemental Figure 5

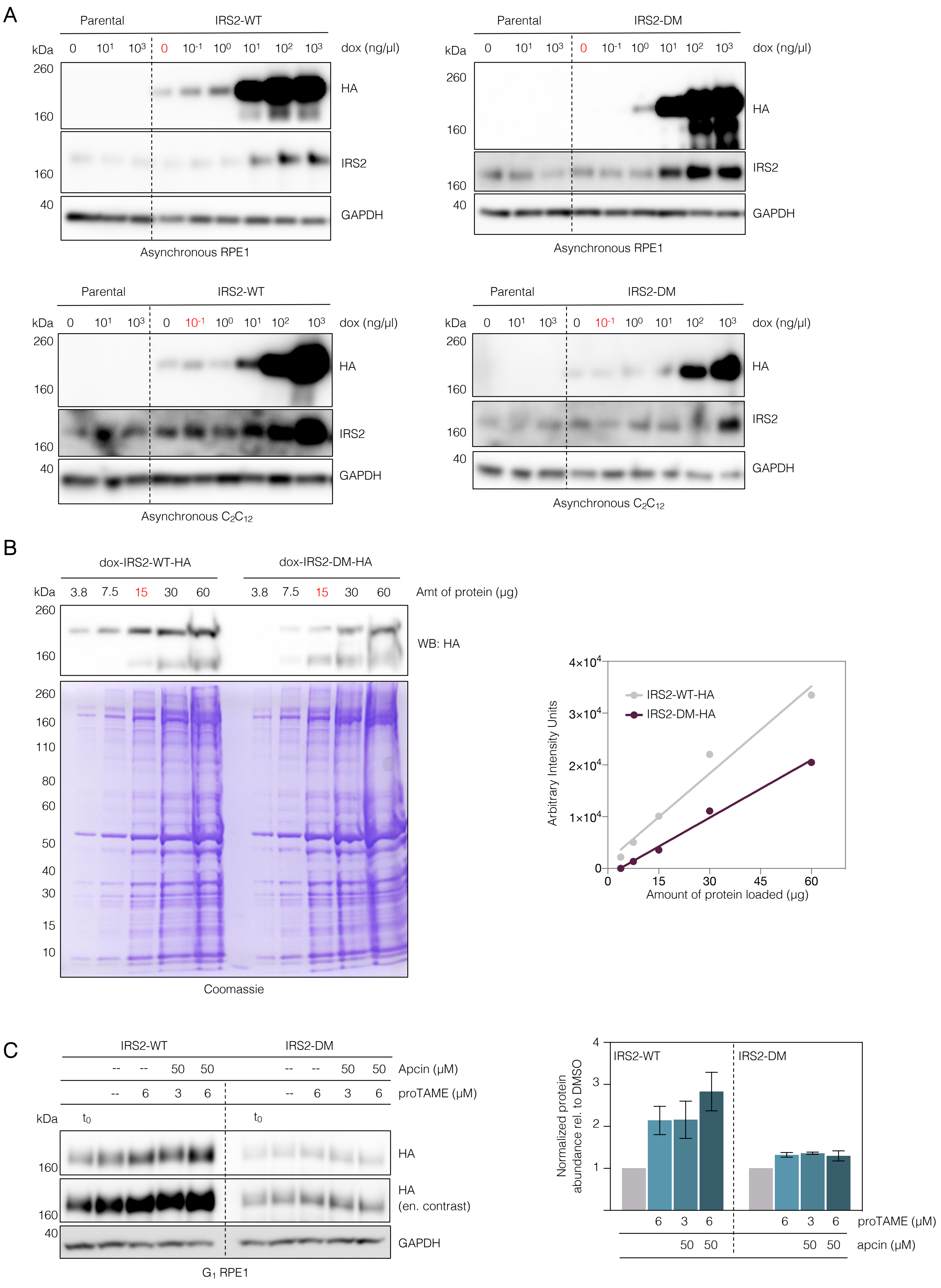

Supplemental Figure 6

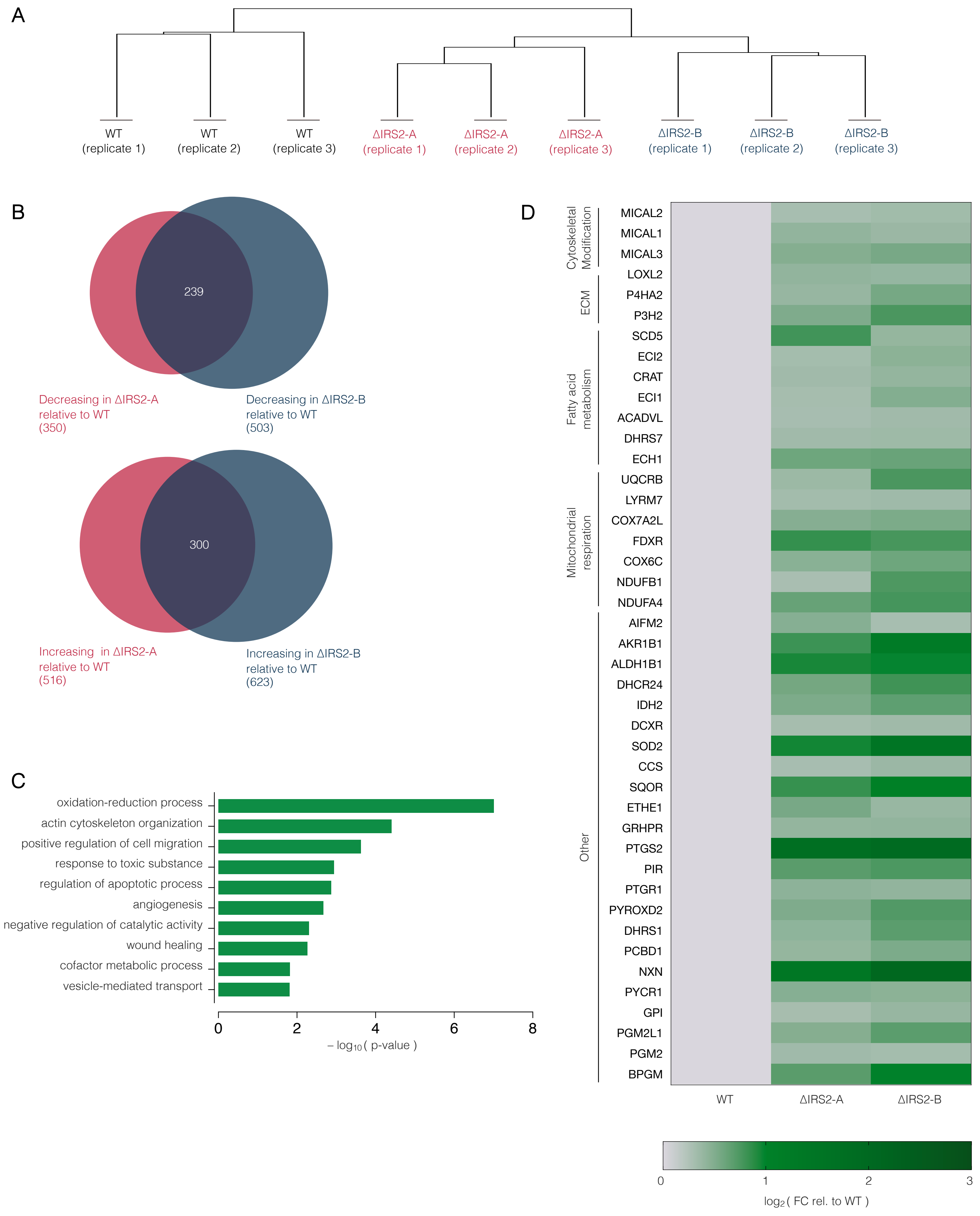

Supplemental Figure 7

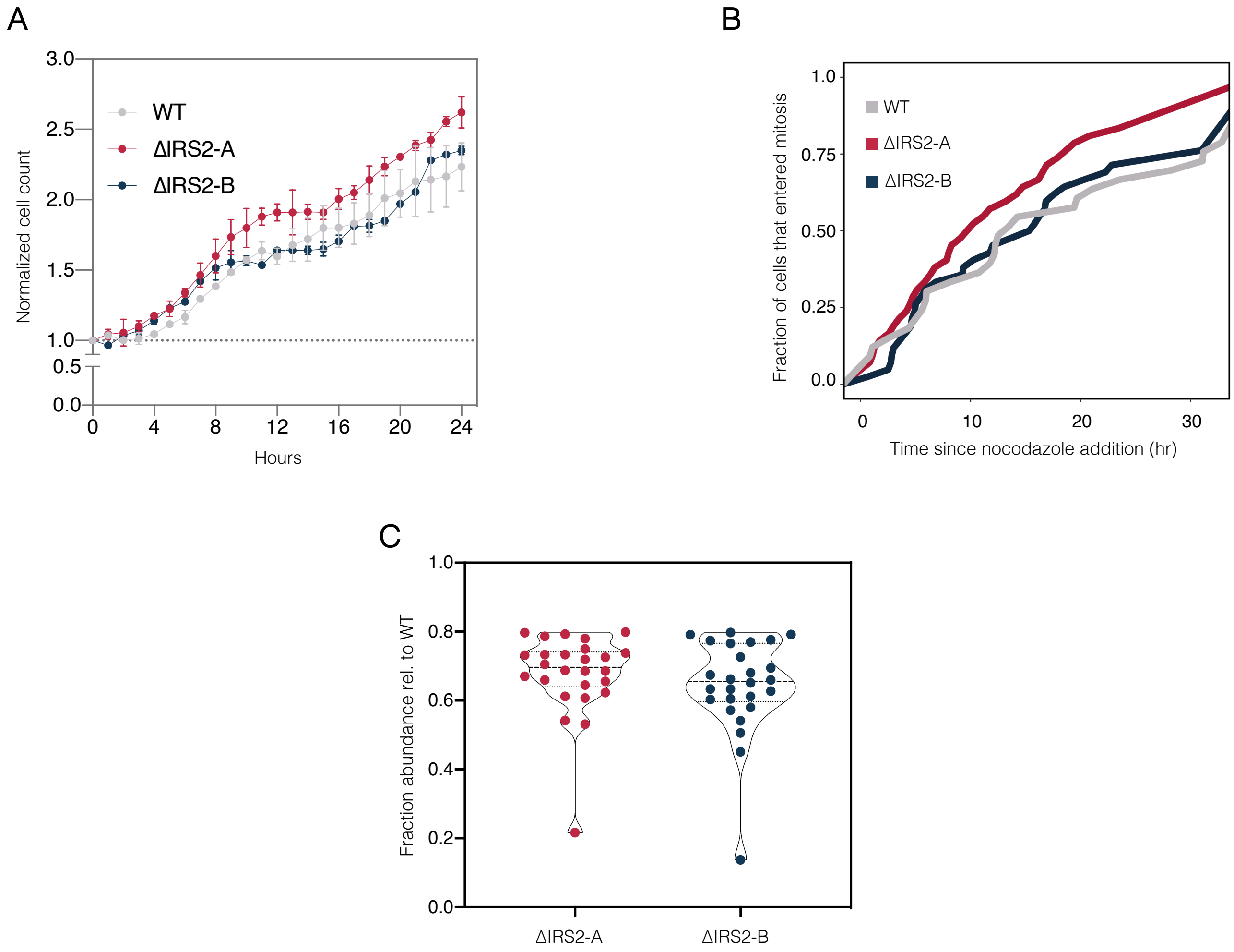

Supplemental Figure 8

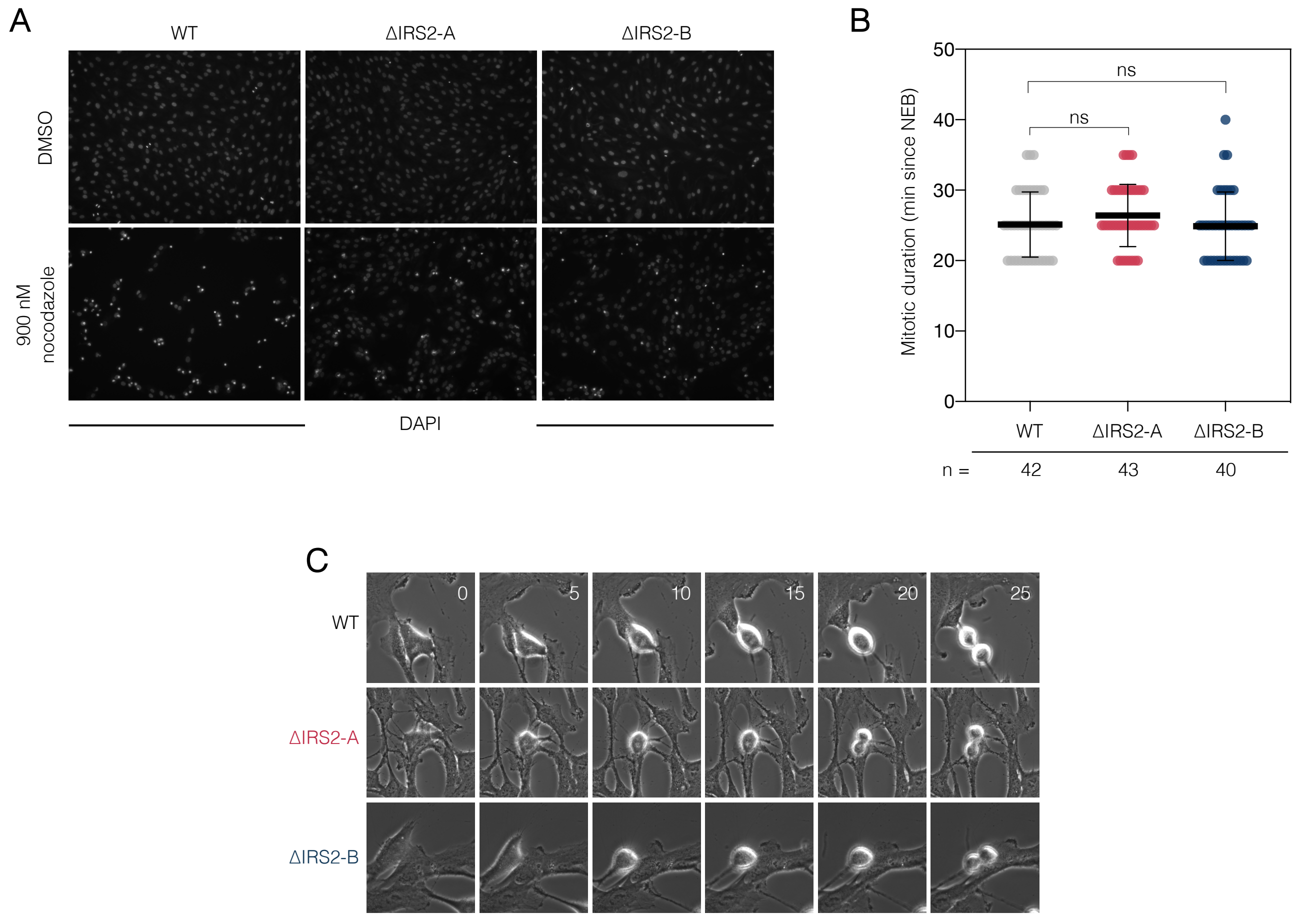

Supplemental Figure 9

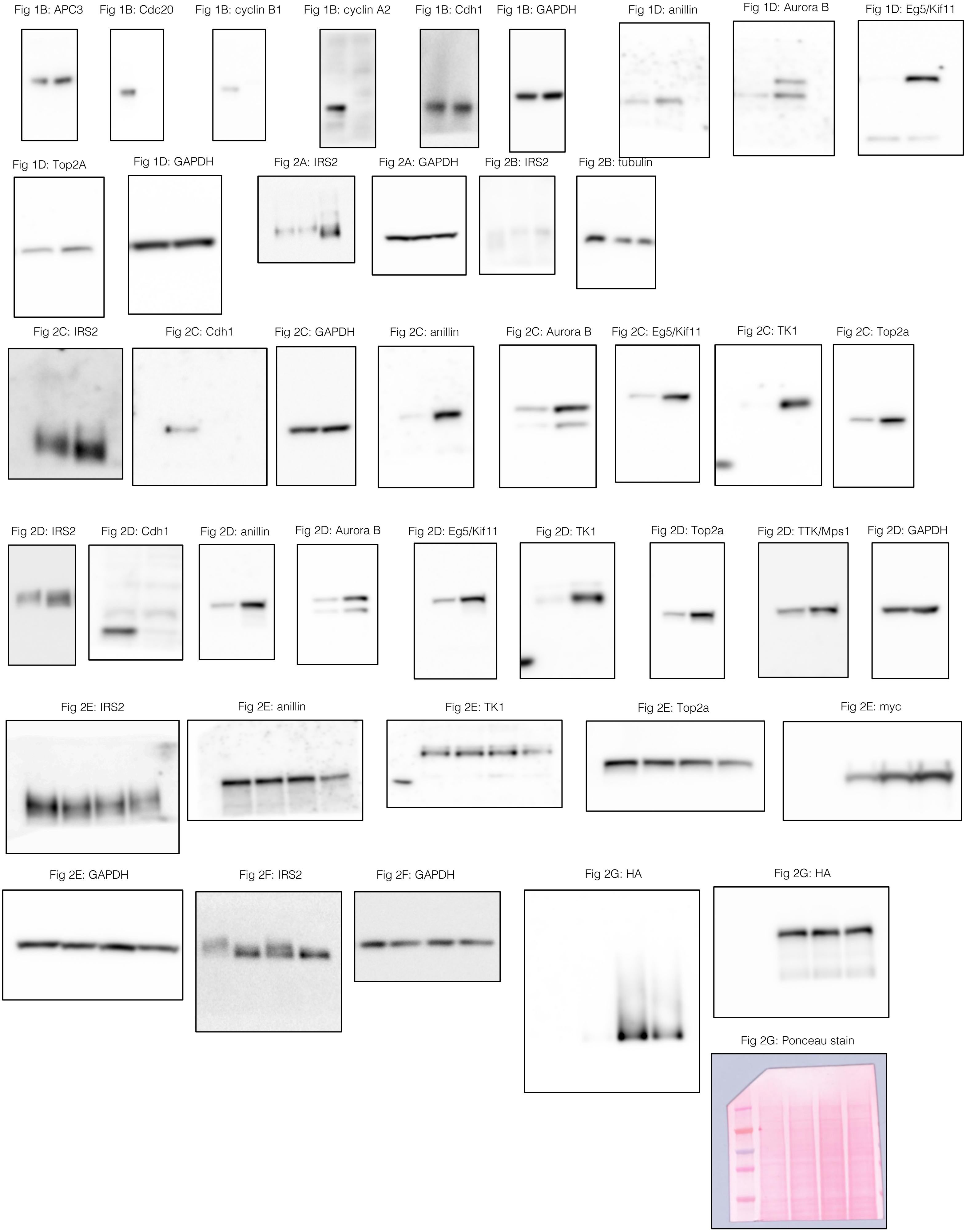

Supplemental Figure 10

Fig 3A: IRS2

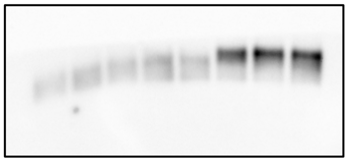

Fig 3: APC3

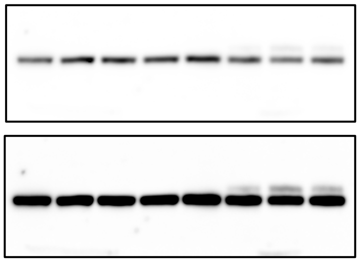

enhanced contrast

Fig 3A: Cyclin B1

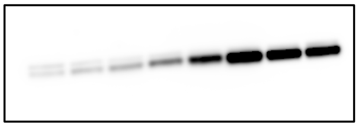

Fig 3A:GAPDH

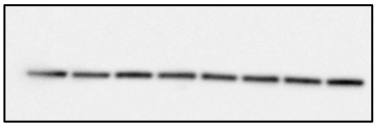

Fig 3B: IRS2

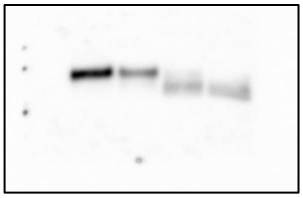

Fig 3B: APC3

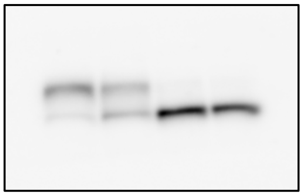

Fig 3B: Cyclin B1

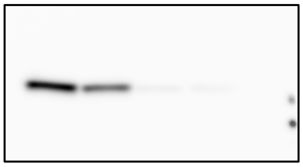

Fig 3B: GAPDH

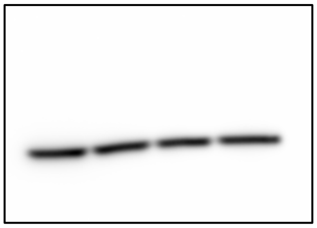

Fig 3C: IRS2

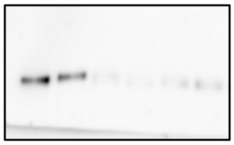

Fig 3C: APC3

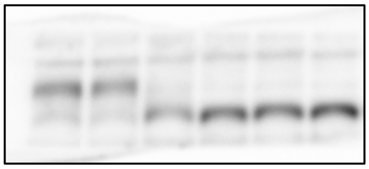

Fig 3C: Cyclin B1

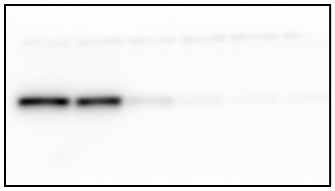

Fig 3C: GAPDH

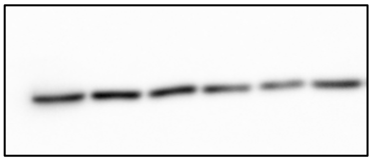

Supplemental Figure 11

Fig 4B: HA

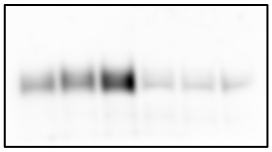

Fig 4B: tubulin

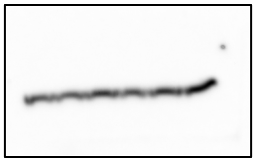

Fig 4C: HA

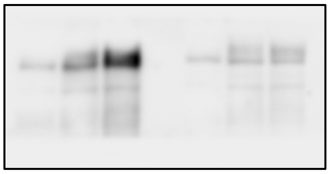

Fig 4C: tubulin

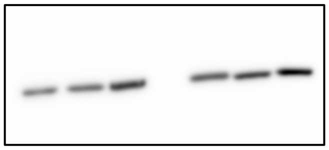

Fig 4D: HA

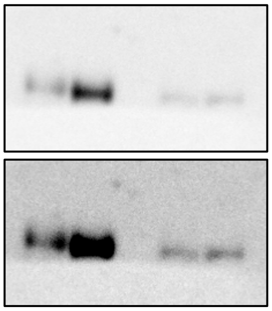

enhanced contrast

Fig 4D: Cdh1

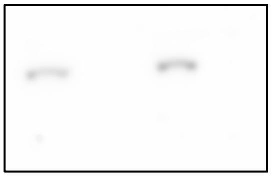

Fig 4D: vinculin

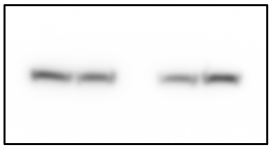

Fig 4E: HA

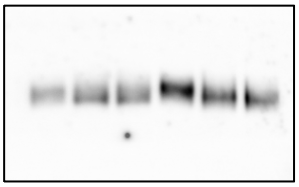

Fig 4H: IRS1

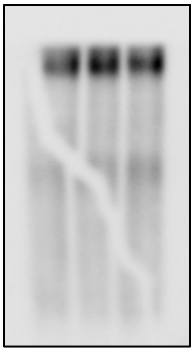

Fig 4H: GAPDH

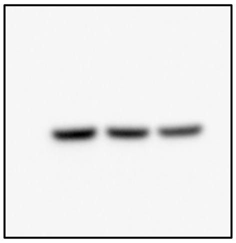

Fig 4E: Cdh1

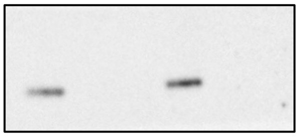

Fig 4E: GAPDH

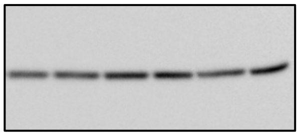

Fig 4I: IRS1

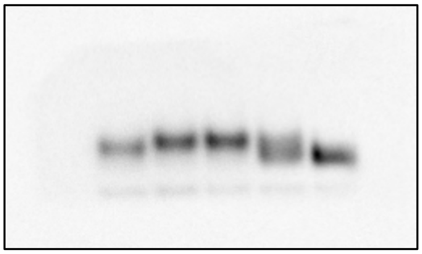

Fig 4I: Cyclin B1

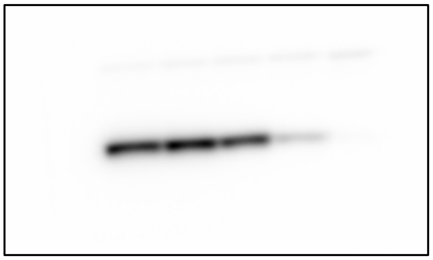

Fig 4I: Cdc20

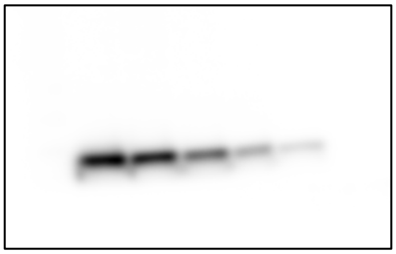

Fig 4I: GAPDH

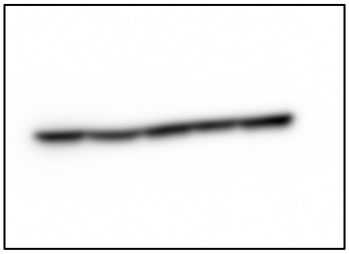

Fig 5A: IRS2

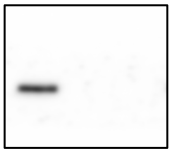

Fig 5A: vinculin

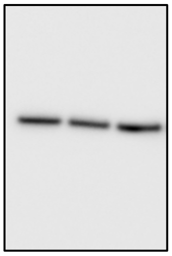

Supplement: Supplementary file 1 [file mmc1.zip › mmc1/159856_1_supp_540476_qb9qfn.pdf]
